# A core-shell-type nanosystem promotes diabetic wound healing through Photothermal-responsive release of transforming growth factor β

**DOI:** 10.1186/s12951-024-02675-2

**Published:** 2024-07-30

**Authors:** Jinfei Hou, Junjin Jie, Xinwei Wei, Xiangqian Shen, Qingfang Zhao, Xupeng Chai, Hao Pang, Zeren Shen, Jinqiang Wang, Linping Wu, Jinghong Xu

**Affiliations:** 1 Department of Plastic Surgery, The First Affiliated Hospital, Zhejiang University School of Medicine, Hangzhou, Zhejiang 310003 China; 2 Center for Chemical Biology and Drug Discovery, Guangzhou Institute of Biomedicine and Health, Chinese Academy of Sciences, Guangzhou, 510530 China; 3 Department of Plastic Surgery, Union Hospital, Tongji Medical College, Huazhong University of Science and Technology, Wuhan, 430022 China; 4 Key Laboratory of Advanced Drug Delivery Systems of Zhejiang Province, College of Pharmaceutical Sciences, Zhejiang University, Hangzhou, 310058 China; 5 Department of Orthopedic Surgery, The Second Affiliated Hospital, Zhejiang University School of Medicine, Hangzhou, 310009 China; 6 Key Laboratory of Immune Response and Immunotherapy, Guangzhou Institutes of Biomedicine and Health, Chinese Academy of Sciences, Beijing, China

**Keywords:** Graphdiyne, Photothermal-responsive, Transforming growth factor β, Diabetic wounds

## Introduction

Diabetic wounds are the most common complication of diabetes, contributing to severe health costs and economic losses [1]. Over 1 million patients with diabetes present with diabetic wounds [2, 3]. Therefore, developing new wound dressings is essential for treating diabetic wounds [4]. However, treating diabetic wounds remains challenging because a high-glucose microenvironment can further induce bacterial breeding, resulting in infection-related complications [2, 5]. Microangiopathy and revascularization defects can also negatively influence healing [6, 7]. The infections and defects of vessels might further trigger a chain of adverse reactions, such as inhibition of cell proliferation and migration [8, 9].

Transforming growth factor β (TGF-β) is a cell factor that potentially helps wound healing [10]. It promotes the growth and migration of epidermal and vascular endothelial cells [11]. It also promotes angiogenesis and consequent blood flow to wounds by stimulating the local release of other growth factors [12]. Furthermore, topical use of medicated wound dressings helps avoid potential damage to other tissues in the body. Therefore, TGF-β is an excellent drug candidate for the local treatment of diabetic wounds [13]. However, there are limitations; cellular factors are likely to degrade in vivo and cannot be sustained for a long time, while diabetic wound healing is chronic and continuous [14]. Additionally, TGF-β lacks the antibacterial properties that are essential for diabetic wound healing [15].

To overcome these difficulties, drug-delivery nanosystems may be used to construct appropriate carriers for TGF-β to cure diabetic wounds. The stimulus-response nanosystems (especially the light-response nanosystem) have been widely used to treat wounds [16]. Drug release could be controllable with an “ON/OFF” switch for light, and the associated temperature rise could result in bacterial death owing to the photothermal effects of these nanosystems. Nevertheless, the high temperature could also deconstruct the molecular structures containing loaded cell factors and harm the surrounding tissues [17, 18]. It is essential to develop thermosensitive nanosystems to achieve antibacterial effects at a safe temperature range of < 50 °C [19, 20].

Consequently, the core-shell-type is a common method to construct scaffolds in regenerative medicine [21] and was applied in this study. Graphdiyne (GDY) was chosen as the core part owing to its stability and biocompatibility reported in our previous study [22]. Most importantly, GDY nanoparticles (NPs) may possess the ability to damage the cell wall of bacteria and produce tiny amounts of reactive oxygen species (ROS), similar to other carbon-based two-dimensional material [23]. It is reasonable to consider that it might be a proper antibacterial and photothermal system functional at relatively safe temperatures. However, the properties to load the drug and kill bacteria in GDY NPs have not been widely surveyed. Thus gelatin, a natural biocompatible material, was chosen to cover the surface of the GDY nanoparticles [24]. The coverage of gelatin could modify the GDY nanoparticles from anion to cation, which might make drug-loading of TGF-β feasible via charge self-assembly [25]. Most importantly, gelatin turns from a gel state to a fluid state at the temperature above 30 °C [26]. This makes it possible for GDY/G NPs (GDY NPs covered with gelatin) to release TGF-β encapsulated in a gelatin layer responsive to photothermal stimulus without affecting its bioactivity owing to the safe state-change temperature [27]. Besides, this photothermal stimulus drug delivery nanosystem serves the purpose to controllably release drugs, avoiding unnecessary loss of drugs and exerting long-term drug release effects [28].

In this study, we propose a near-infrared (NIR)-responsive nanosystem based on GDY and gelatin and loaded with TGF-β. The GDY/G@TGFβ NPs were further encapsulated in biocompatible polyethylene glycol diacrylate (PEGDA) hydrogels with good mechanical properties. The drug release curve and capacity of the hydrogels to promote cell migration and proliferation, antibacterial effects, and revascularization were determined. Finally, full-thickness wounds in diabetic mouse models were constructed to investigate the ability of the PEGDA-GDY/G@TGFβ hydrogels to promote diabetic wound healing and tissue regeneration.

## Materials and methods

### Synthesis of PEGDA-GDY/G@TGFβ hydrogels

PEGDA-GDY/G@TGFβ hydrogels were synthesized by the method of charge self-assembly. GDY (#102,499, XFNano, China) nanoparticles at concentrations of 0.05%, 0.1% and 0.5% were prepared as described before [22]. The gelatin (ThermoFisher Scientific, USA) was dissolved in the GDY nanoparticles solution above and the concentration of gelatin in the mixture solution was controlled at the level of 0.1%. GDY nanoparticles were incubated at 4 °C for approximately 10 min with gentle shaking. After centrifugation at 15,000 rpm at 25 °C for 10 min, the supernatant is discarded. The deionized water was used to wash the redundant gelatin and then TGF-β1 (#80,116, Sino Biological, China) at the concentration of 100 µg/L was dissolved into the dispersion liquid. After centrifugation as above, the GDY/G@TGFβ solution was prepared. The 10 mg PEGDA (#729,094, Sigma-Aldrich, USA) and 2 ∼ 3 mg lithium pheny l-2,4,6-trimethylbenzoyl phosphinate (LAP) (#85073-19-4, EFL, China) was added to 1 mL solution above and completely dissolved. To construct the hydrogels, blue light irradiation was performed for at least 20 s. For sterilization, GDY powder was immersed in 75% ethyl alcohol for 12 h and then washed with sterile water and collected via centrifugation. The other non-sterile materials were sterilized with ethylene oxide. The fabrication of hydrogels was processed in a clean bench. Besides, to measure the loading efficiency of TGF-β, GDY/G@TGFβ nanoparticles were washed in PBS and centrifuged again to remove the unloaded TGF-β, and this process was repeated three times. The amount of unloaded TGF-β (in supernatants after each washing step) was quantified using the enzyme-linked immunosorbent assay (ELISA) kit (EMC107b.96, NeoBioscience, China). By subtracting unloaded TGFβ from total initial TGFβ, the loading efficiency is defined as (total amount-unloaded amount)/total amount. The loading efficiency of TGF-β in the hydrogel was calculated to be 57.60 ± 7.79%.

### Characterization and morphology of PEGDA-GDY/G@TGFβ hydrogels

To test the dispersity and morphology, PEGDA-GDY/G@TGFβ solution before crosslinking was characterized with dynamic light scattering (DLS) and transmission electron microscopy (TEM). The 100 µL sample solution was diluted into 1 mL deionized water and then the solution was transferred to the corresponding measuring tube for measurement of particle size and zeta potential in NanoSight (Malvern Panalytical, British). After ultrasonic dispersion for at least 60 min, the sample solution was dripped onto a copper grid. Then the redundant liquid was dried and samples were observed by HT7700 microscope (Hitachi, Japan). Besides, Cryoelectron microscopy (Cryo-EM) (S-4800, HITACHI, Japan) was utilized to view the normal surface morphology of hydrogels. Briefly, hydrogels were loaded on a low-temperature sample carrier (Quaroum PP3000T) and then fixed in a cryo-specimen holder The temperature was placed in cold nitrogen (−120 °C), and then immediately transferred to the cryo-stage. To remove the ice crystals, the temperature was raised to 90 °C and after that, the samples were cooled to −180 °C for stabilization. The pore sizes were analyzed by the software Image J (America) on the surface images and the frequency distribution histograms were plotted according to the data. The hydrogels were cut into slices at a diameter of 10 mm and immersed into simulated body fluids (BZ310, Biochemazone, America). After several hours, the immersed hydrogels were weighed. The hydrogels after being immersed for 24 h were performed with tensile experiments described below.

### Mechanical properties of PEGDA-GDY/G@TGFβ hydrogels

#### Tensile, compressive and adhesion tests

Gels were prepared as strips with dimensions (30 mm×10 mm×2 mm) for tensile measurement. The direction of stretching was along the long axis of the hydrogels and the stretching speed was set as 10 mm/min. The constant stretching was kept until the hydrogels ruptured. The tensile modulus (the tangent slope of the stress-strain curve), ultimate strength (strain level at failure) and rupture strain (stress at failure) were calculated according to the results. Besides, for compressive tests, hydrogels were cut into slices at a diameter of 10 mm and thickness of 2 mm the compression speed was set as 5 mm/min. Likewise, the compressive modulus was calculated. Besides, hydrogels were prepared as slices with a diameter of 20 mm and fixed to one side of a mechanical arm. The strength to pull off samples from the platform was measured. The experiments above were performed *via* an all-electric dynamic test instrument (Instron, British). All of the above data and plots were obtained using OriginPro software (OriginLab, America).

### Rheological tests

The hydrogels were cut into uniform disks with a diameter of 10 mm and thickness of 1 mm and the rheological tests were performed by using a modular compact rheometer (MCR102, Anto Paar, Germany). The hydrogels were cut into uniform slices at the diameters of 10 mm and 2 mm and the surface of the hydrogels was kept smooth. The amplitude sweep (AS) of hydrogels was tested from 0.01 to 1000% at a constant frequency of 1 Hz and the frequency sweep (FS) of hydrogels was tested from 0.1 Hz to 10 Hz [29].

### Photothermal effects of PEGDA-GDY/G@TGFβ hydrogels

The hydrogels were placed in the culture dish at room temperature and irradiated with an 808 nm laser for 2 min. The infrared thermal images were captured using an IR camera, and the temperature was recorded at 0 s, 5 s, 10 s, 20 s, 30 s, 45 s, 60 s and 120 s, respectively.

### Drug release of PEGDA-GDY/G@TGFβ hydrogels

The hydrogels were immersed into the 2 mL PBS in tubes, and then 1mL solution was extracted at different time points and 1 mL PBS were supplemented into the tubes. For PGGT(PEGDA-GDY/G@TGFβ)-NIR (+) group, the hydrogels were irradiated for 30 s before extraction every day until the release experiment ended. After sample collection, the concentration of TGFβ in the solution was tested with an ELISA kit. For further experiments, the TGF-β solution at the concentration of 100 µg/L was processed at different temperatures for different times and irradiated with NIR for different times. The macro views of solution after procession with ELISA kit were captured and relative amount of TGF-β in solution were measured at an absorbance of 450 nm.

### In vitro biocompatibility tests of PEGDA-GDY/G@TGFβ hydrogels

Gel disks with diameters of 10 mm and height of 5 mm were sterilized and immersed into culture medium. Human dermal fibroblasts (HDFs) (ATCC, USA) were applied in cytological experiments in vitro. HDFs were cultured in low glucose medium (#C11885500BT, Gibco, USA) supplemented with 10% fetal bovine serum (FBS, #10,099,141, Gibco, USA) and 1% penicillin/streptomycin (P/S, #15,070,063, Gibco, USA) at 37 °C under 5% CO_2_. The medium was exchanged every 2 days, and HDFs were passaged prior to reaching 80% confluence. Cell implantation in hydrogels was performed by the “bottom-up” strategy, which made sure that cells were encapsulated in hydrogels and sharply reduced the incidence of cell loss. Briefly, HDFs were collected and resuspended evenly in sterilized and uncrosslinked hydrogel solution. The concentration of cell suspension in the hydrogels was 106/mL. After the process of crosslinking, the hydrogels loaded with HDFs were transferred to the incubator. The fluorescein diacetate (FDA)/propidium iodide (PI) staining and CCK8 assay were performed as mentioned previously [22]. The viability of HDFs in hydrogels was calculated according to fluorescence staining images *via* the software Image J. The cytoskeleton was stained with phalloidin-FTIC conjugates at a concentration of 0.5 mg/mL.

### Cell migration experiments affected by hydrogels

For cell migration experiments, the extract liquor was prepared by medium immersed with hydrogels for 24 h. HDFs were cultured in a 6-well plate and reached 80% confluence. The sterilized tips were applied to make the scratch through the cell colony. Then the scratch images were captured at 0 h, 8 h, 16 h and 24 h, respectively. The wound width-time curve was plotted and the linear regression analysis was utilized to calculate the rate of closure.

### Assessment of angiogenesis in vitro of hydrogels

HUVECs were cultured in RPMI 1640 medium (#C11875500BT, Gibco, USA) supplemented with 10% fetal bovine serum and 1% penicillin/streptomycin at 37 °C under 5% CO_2_. Then HUVECs were collected and resuspended at the concentration of 10^6^/mL. After implantation in gel disks as described before, the cytoskeleton of HUVECs was stained by the solution of phalloidin (p5282, Sigma-Aldrich, USA) at the concentration of 0.1 mg/mL at the different time points of 24 h and 48 h. Then the number of the tube was analyzed and the mean OD values of CD31 (/pixel) were calculated in Image J software.

### Antibacterial effects in vitro of hydrogels

Escherichia coli (*E. coli*, Gram-negative, ATCC25922, USA), Pseudomonas aeruginosa (*P. aeruginosa*, Gram-negative, ATCC27853, USA), Staphylococcus aureus (*S. aureus*, Gram-positive, ATCC43300, USA), and methicillin-resistant Staphylococcus aureus (*MRSA*, ATCC43000, USA) were used to perform antibacterial tests. Single colonies grown on Luria-Bertani (LB) plates were inoculated into LB broth medium at 37 °C with shaking at 200 rpm overnight until logarithmic growth (OD590 = 0.8). Bacterial liquid was centrifuged, after which the supernatant was discarded. The bacteria were collected suspended in LB broth medium, and the final bacterial concentration was 1.0 × 10^6^ CFU/mL. The bacterial numbers were calculated from the measurement of absorbance at 590 nm using a UV-Vis spectrophotometer. Then experimental conditions were then set up with a group containing 900 µL of 10^4^ CFU/mL of bacterial suspension, prepared as above, together with (1) 100 µL PBS, (2) PEGDA hydrogels, (3) PEGDA-GDY hydrogels, (4) PEGDA-GDY/G, and (5) PEGDA-GDY/G@TGFβ, and the groups after irradiated by an 808 nm laser (500 mW/cm^2^) for 30s. These tubes were then incubated for 2 h at 37 °C, after which the bacterial suspensions were coated on LB agar plates for incubation overnight and colonies were counted. Besides, the experiments of the inhibitory zone were performed as follows. The hydrogels were put on the LB agar plates inoculated with various bacteria, and the images were captured at the time point of 24 h after the hydrogels were irradiated with NIR.

For antibacterial mechanism experiments, the ROS fluorescence was stained with antibodies described in Table S3 and the intensity of ROS fluorescence was measured *via* ZEISS software (German). The bacteria of *S. aureus* at the concentration of 1 × 10^8^ CFU/mL were incubated in hydrogels. After NIR-irradiation for 30 s, the bacterial suspensions were centrifuged and filtered with a 0.22 μm membrane. The remained supernatant was tested as the leakage of intracellular DNA and RNA by measuring the OD_260_ value of each sample using UV-vis measurement. Besides, the HDFs implanted in hydrogels after 48 h were collected and the IL-6 were measured via IL-6 ELISA kit (RAB0306, Sigma-Aldrich, USA). ROS levels in bacteria were measured using the ROS probe (DCFH-DA, HARVEYBIO, China).

### In vivo implantation evaluation on the diabetic wound mouse model

All procedures on animals were processed under the National Institute of Health’s Guidelines for the Care and Use of Laboratory Animals and in accordance with the Consent Form for Ethical Review of Animal Experiments (No. N2019066). The C57/8BL6 male mice were intraperitoneally injected with streptozocin (STZ, 50 mg/kg) (HARVEYBIO, China) solution once a day for five days. Diabetic wound model animals were selected from mice with fast glucose levels > 11.1 mmol/L for at least two detections. Thirty mice with diabetes were randomly divided into five groups of PEGDA, PEGDA-GDY, PEGDA-GDY/G, PEGDA-GDY/G@TGFβ and PGGT-NIR (+). All operations were performed by the same surgeon under general anesthesia with 2% isoflurane inhalation. After the mice were completely unconscious, the dorsal hair was shaved and applied with a depilatory cream. A full-thickness skin in 8 mm diameter was excised on the dorsa of mice mentioned above to make a diabetic wound mouse model. Then sterilized hydrogels were prepared as slices with an inner diameter of 8 mm and height of 2 mm and fixed on the wounds. The operation region was captured at the time point of 0, 1, 3, 7 and 14 days and the wounds were harvested for 14 days after carbon dioxide asphyxia. Besides, the wound of PGGT-NIR (+) group was irradiated with an NIR laser for 30 s every day. For histological staining and immunofluorescence staining, the wound skin was fixed and embedded in paraffin and samples were stained according to antibodies shown in Table S3. Besides, the amount of collagen and elastin was measured using the Total Collagen Assay kit (BioVision, USA) and the Fastin Elastin Assay kit (Biocolor, UK). Blood chemistry analysis was performed using Vetscan VS2 (Abaxis) and kits (Bioassay Systems, CA, USA). The antibodies utilized were shown in Table S3.

### Statistical analysis

All results were shown as mean ± SD. One-way analysis of variance (ANOVA) or two-way ANOVA were performed to analyze differences among three or more groups, followed by Tukey, Sidak or Bonferroni correction and the two-tailed Student’s test was performed to analyze the statistical significance between two group. Statistical significance was set at *P* < 0.05 and non-significant results were recorded as N. S.

## Results

### Fabrication and characterization of PEGDA-GDY/G@TGFβ hydrogels

The particle size of a 0.1% GDY solution was close to 100 nm and the polydispersity index (PDI) was much lower than that of the 0.5% GDY solution (Fig. S1), which indicated that nanoparticles at a concentration below 0.5% present more even size and were less prone to aggregation. The amount of drug loading for carrier is also taken into consideration. Theoretically, the higher the carrier concentration, the higher the drug load in most instances [30, 31]. Thus 0.1% GDY concentration was selected for this study rather than 0.05% GDY. Sample solutions from all groups were observed using TEM and analyzed using DLS. A gelatin coating was distinctly observed around the GDY NPs (Fig. S2A–C). The gelatin coating caused the GDY NPs to shift from anionic to cationic and made it possible to load negatively charged TGF-β. The process of loading TGF-β and electrostatic self-assembly did not significantly affect the particle sizes and the zeta potentials of the NPs were slightly decreased after TGF-β loading. Besides, the results in Fig. S2D revealed that the addition of gelatin and TGF-β would not lead to aggregation of nanoparticles.

**Fig. 1:**
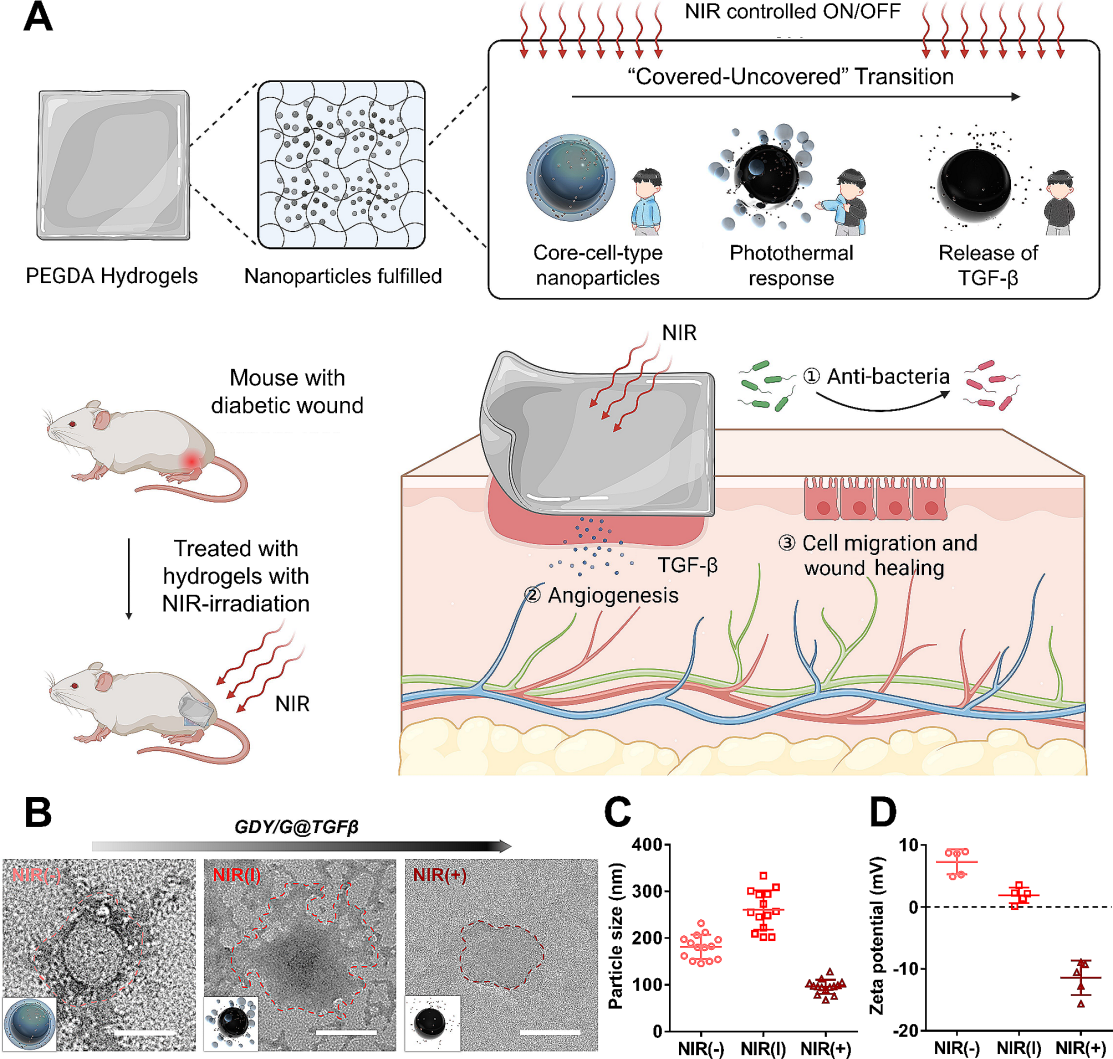
The process of drug release of PEGDA-GDY/G@TGFβ. **(A)** Schematic of transformation after NIR-irradiation for PEGDA-GDY/G@TGFβ hydrogels. **(B)** TEM images of uncross-linked PEGDA-GDY/G@TGFβ solution in the process of NIR-irradiation. Scale bar, 100 mm. NIR (-): without NIR-irradiation. NIR (|): with NIR-irradiation for 30 s. NIR (+): with NIR-irradiation for 120 s. Dash line: the edge of nanoparticles. **(C)** The particle size of uncross-linked PEGDA-GDY/G@TGFβ solution in the process of NIR-irradiation (*n* = *14*). **(D)** Zeta potential of uncross-linked PEGDA-GDY/G@TGFβ solution in the process of NIR-irradiation (*n* = *5*)

Figure 1A shows the dynamic procedure of the NIR irradiation of the PEGDA-GDY/G@TGFβ solution before cross-linking. Due to the photothermal effects of GDY, the outer gelatin layer of nanoparticles was gradually lost, and the loaded TGF-β was released (Fig. 1B). The particle sizes of PEGDA-GDY/G@TGFβ group significantly decreased and their stability declined after NIR irradiation (Fig. 1C and Fig. S3). The zeta potential of PEGDA-GDY/G@TGFβ group significantly decreased after irradiation (Fig. 1D), further confirming its “covered-uncovered” transition.

**Fig. 2:**
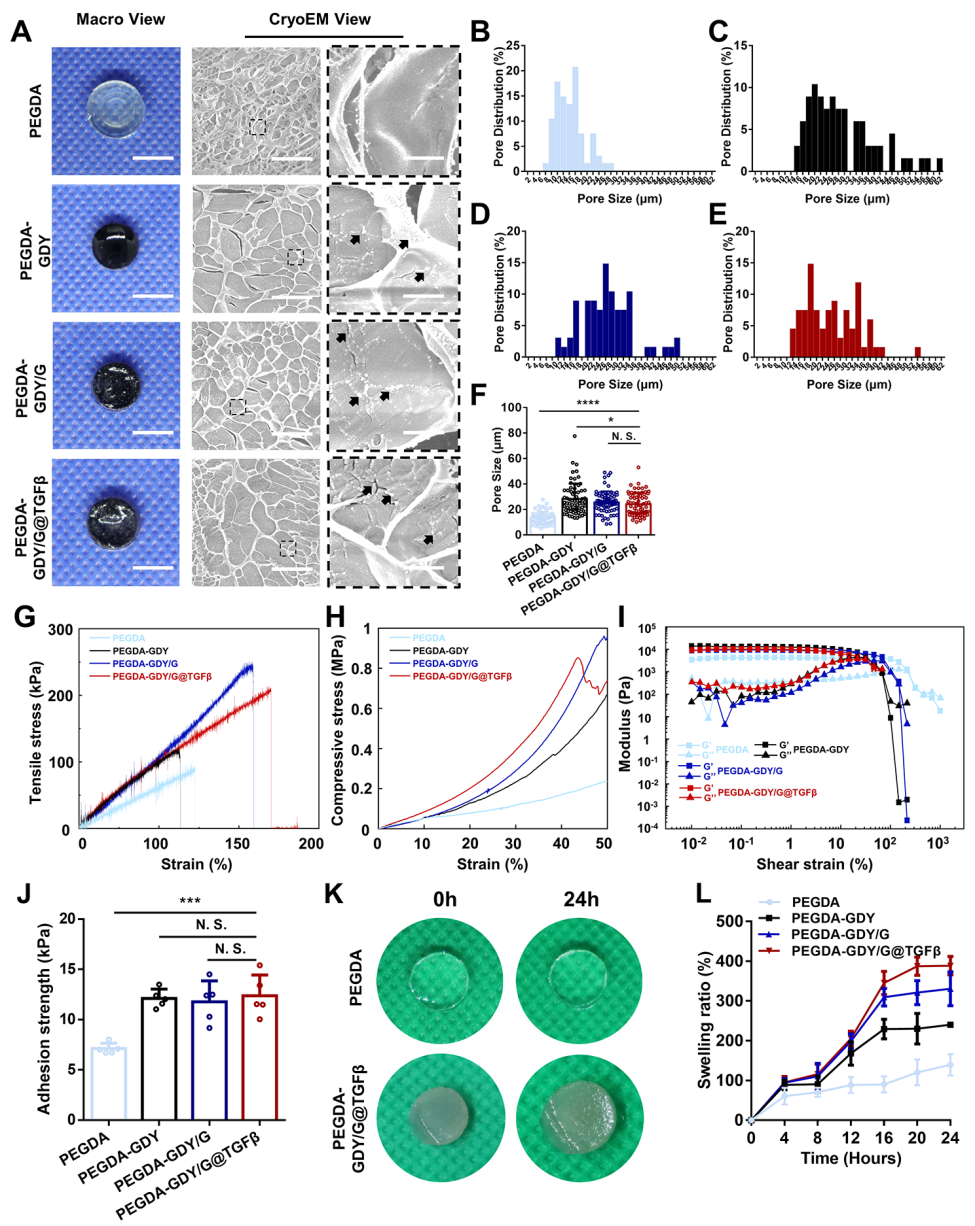
Morphological and photothermal characteristics of different hydrogels. **(A)** Macro views and CryoEM images of different hydrogels. The distribution of pore size of **(B)** PEGDA, **(C)** PEGDA-GDY, **(D)** PEGDA-GDY/G and **(E)** PEGDA-GDY/G@TGFβ hydrogels. **(F)** The pore size of hydrogels. n = 68, ****P < 0.0001, * P < 0.05, by one-way ANOVA with Tukey correction. **(G)** The tensile stress-strain curve of hydrogels. **(H)** The compressive stress-strain of hydrogels. **(I)** The shear strain of hydrogels at a constant frequency of 1 rad/s. G’: storage modulus, G’’: loss modulus. **(J)** The adhesion strength of different hydrogels after NIR-irradiation. *n* = *5*, *** *P* < *0.001*, by one-way ANOVA with Tukey correction. **(K)** The macro view of different hydrogels after immersion in the simulated body fluid. **(L)** The swelling ratio of different hydrogels after immersion in the simulated body fluid. *n* = 5

Cryoelectron microscopy was used to study the surface structures of the hydrogels after cross-linking. The porous morphologies of hydrogels are shown in Fig. 2A. According to the analysis of pore size distribution in Fig. 2B–F, the pore sizes of PEGDA-GDY, PEGDA-GDY/G, and PEGDA-GDY/G@TGFβ were close to 30 μm, which has been reported to be beneficial for tissue regeneration [7]. Interestingly, the addition of GDY had an evident impact on the pore sizes of the hydrogels, and different concentrations of GDY NPs affected the porosities of the hydrogels in different ways (Fig. S4). The GDY NPs also could be observed to uniformly distribute in the hydrogels.

Most hydrogel wound dressings have brittle mechanical properties when used on dynamic wounds, especially for joints and axilla, leading to wound infection caused by unnecessary tearing [32]. Thus, it is important for hydrogel wound dressings to have high mechanical strength and sustained stability [33]. For most NPs-incorporated hydrogels, their toughness and strengths change distinctly. The GDY NPs in the PEGDA hydrogels enhanced the ultimate strengths, rupture stains, and tensile moduli (Fig. 2G and Fig. S5A–C). The PEGDA-GDY/G@TGFβ could withstand a tensile stress of 220.69 ± 20.82 kPa and a tensile strain of 146.69 ± 21.68%. The compressive modulus of PEGDA-GDY/G@TGFβ also increased after being mixed with the GDY NPs (Fig. 2H and Fig. S5D). To examine the hydrodynamic characteristics of the materials, frequency and amplitude sweeps were performed (Fig. 2I and Fig. S6), and it was confirmed that the GDY-incorporated hydrogels possessed better shear resistance [22]. There was no significant difference between PEGDA-GDY/G group and PEGDA-GDY/G@TGFβ group, illustrating that the process of drug loading would not cause the weaken of shear mechanics. We inferred that the enhanced mechanical properties were correlated to the mixture of GDY nanoparticles. Previous studies have reported that the introduction of nanoparticles such as graphene oxide and GDY enhances the hydrogel network and enriches the entanglement of macromolecular chains, effectively dissipating the energy when stretching, compressing, and shearing [33]. Besides, it also could be inferred from Figs. S5 and S6 that the drug loading of TGF-β did not affect the mechanical properties of hydrogels such as tensile modulus, compressive modulus, rupture strain, ultimate strength and storage modulus.

Adhesion is an important property for wound dressings because tight adhesion and close coverages decrease the risk of wound infections, which is one advantage of hydrogel dressing compared to traditional dressings [34]. PEGDA-GDY/G@TGFβ hydrogels showed good adhesion strength, approximate to commercial fibrin sealants (Fig. 2J). Additionally, the PEGDA-GDY/G@TGFβ hydrogel maintained excellent adhesion stability after six strip-adhesion tests and the adhesion only slightly decreased, indicating that it possessed repeated and long-lasting adhesion stability (Fig. S7). Furthermore, the capacity of hydrogel dressing to absorb the excessive blood and tissue exudate was beneficial to wound healing [35]. In this research, the swelling ratio of hydrogels indicated that the PEGDA-GDY/G@TGFβ hydrogel showed high water absorbability (388.7 ± 23.09%) after incubation in simulated body fluid, which was much higher than that of the PEGDA hydrogel (Fig. 2K and L). The swelling significantly affected the tensile modulus of PEGDA-GDY/G@TGFβ hydrogel rather than the PEGDA hydrogel (Fig. S8). This might be correlated with the increase in molecular distance and the associated weakening of the hydrogel network [33]. Despite the decline in mechanical properties, hydrogels can still meet the requirements of wound dressing under a favorable physiological environment [34].

**Fig. 3:**
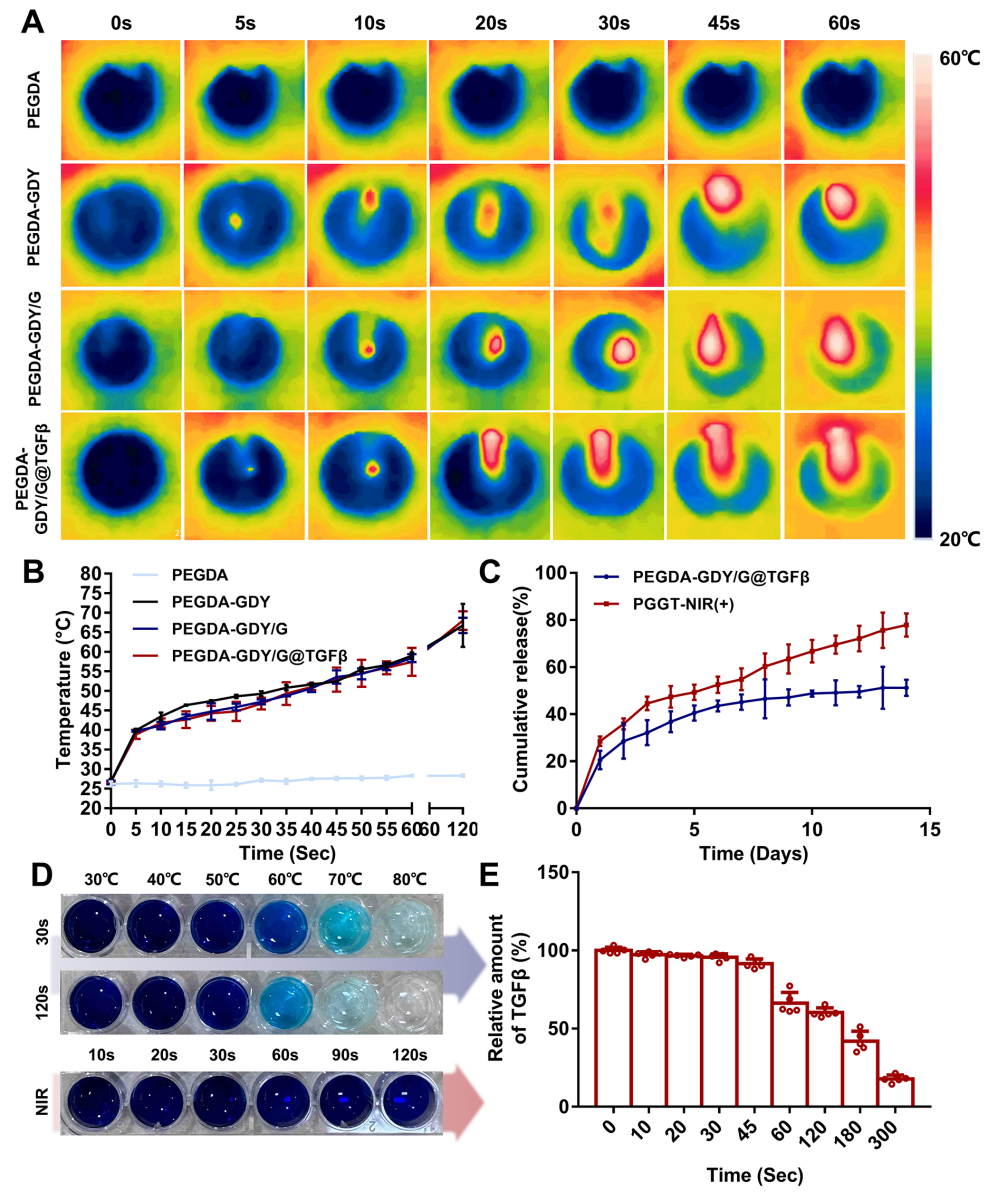
The photothermal effects of different hydrogels. **(A)** Pseudo-color thermal image of different hydrogels with NIR-irradiation at different time points. **(B)** The line chart of temperature changes with time variation for different hydrogels with NIR-irradiation. **(C)** The cumulative release of TGF-β of PEGDA-GDY/G@TGFβ and PGGT-NIR (+). PGGT-NIR (+): PEGDA-GDY/G@TGFβ with NIR-irradiation. **(D)** The staining of TGF-β solution after pure heating and pure NIR-irradiation. **(E)** The relative amount of TGF-β in GDY nanoparticles solution after NIR-irradiation. *n* = xx

The photothermal-responsive properties and stimulus-response drug release characteristics of PEGDA-GDY/G@TGFβ are important for the effectiveness of this nanosystem. Hydrogels containing GDY NPs exhibited a temperature increase after NIR irradiation for a few seconds (Fig. 3A). The temperature-time curve showed that the temperatures of PEGDA-GDY/G and PEGDA-GDY/G@TGFβ increased slower than that of PEGDA-GDY in the early stage. The curves were similar after 45 s, possibly owing to the loss of gelatin layer (Fig. 3B). The irradiation time was controlled to within 30 s to exert the antibacterial effects and ensure that the temperature would not be too high to affect TGF-β activity. Additionally, it can be inferred that PEGDA-GDY/G@TGFβ exhibited time-dependent sustained drug release (Fig. 3C). PGGT-NIR (+) (PEGDA-GDY/G@TGFβ after NIR irradiation) exhibited better drug release than that of PEGDA-GDY/G@TGFβ. The cumulative drug release of PGGT-NIR (+) (77.86 ± 4.92%) was also greater than that of PEGDA-GDY/G@TGFβ (51.20 ± 3.42%). Thus, the rate of change of temperature of the GDY NPs was faster than that of graphene oxide NPs and the targeted temperature was reached in seconds [36]. This property is beneficial for the stability of protein drugs such as cell factors in drug delivery [37].

The change of absorbance showed that a change in conditions results in TGF-β denaturation (Fig. 3D). The temperature increase had more negative effects on TGF-β than NIR-irradiation and NIR-irradiation for a short time (several minutes) barely had any influence on TGF-β. To mimic the variation of TGF-β in NIR-responsive drug release, the concentration of TGF-β solution with GDY nanoparticles was measured at different time points after NIR-irradiation (Fig. 3E). The loss of the amount of TGF-β influenced by the process of NIR-responsive photothermal effects in this study (irradiation for ∼ 30 s) was below 3%, which is negligible. It indicated the eminent properties of this nanosystem as a drug carrier for bioactive drugs that were sensitive to temperature and prone to degradation, such as nucleic acid drugs and protein drugs. The key point is that the NIR-controlled drug release could be achieved in 30 s of irradiation and at a relatively safe temperature (< 50 °C). This is a significant improvement compared to previously described photothermal nanosystems [38].

### In vitro antibacterial effects of PEGDA-GDY/G@TGFβ hydrogels

**Fig. 4:**
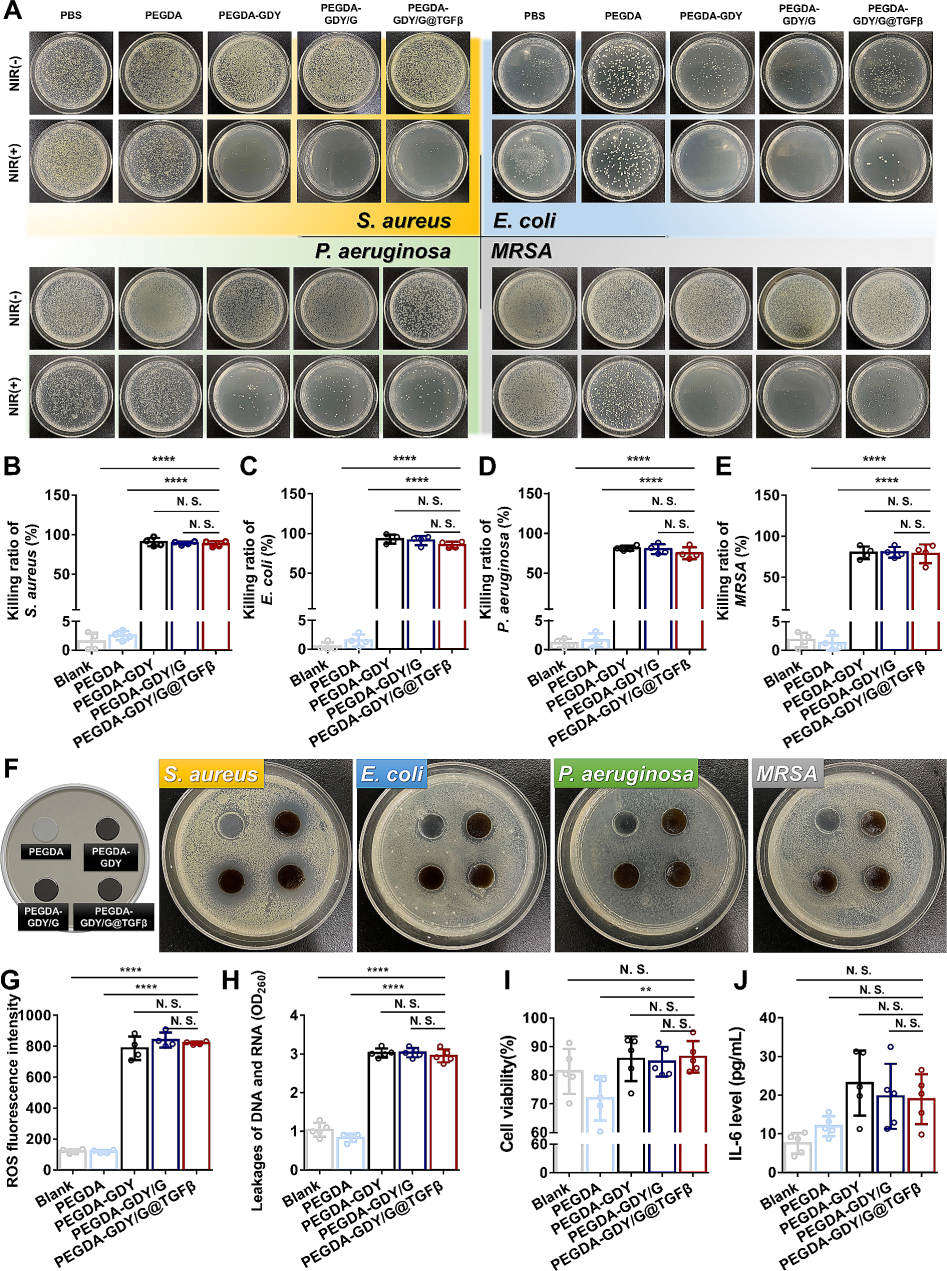
The antibacterial effects properties of different hydrogels in vitro. **(A)** Macro views of agar plates inoculated with *S. aureus*, *E. coli*, *P. aeruginosa* and *MRSA* suspension after immersion with different hydrogels with/without NIR-irradiation. NIR(-): without NIR-irradiation. NIR (+): with NIR-irradiation for 30 s. The killing ratio of **(B) ***S. aureus*, **(C) ***E. coli*, **(D) ***P. aeruginosa* and **(E) ***MRSA* for different hydrogels with/without NIR-irradiation was plotted. *n* = 4, ****P < 0.0001, by one-way ANOVA with Tukey correction. **(F)** The inhibitory zones of *S. aureus*, *E. coli*, *P. aeruginosa* and *MRSA* for different hydrogels with NIR-irradiation at the time point of 24 h. **(G)** The ROS fluorescence intensity of different hydrogels after NIR-irradiation. *n* = 4, ****P < 0.0001, by one-way ANOVA with Tukey correction. **(H)** The leakages of DNA and RNA of *S. aureus* after processing with hydrogels after NIR-irradiation. *n* = 5, ****P < 0.0001, by one-way ANOVA with Tukey correction. **(I)** The cell viability of HDFs implanted in different hydrogels after NIR-irradiation. *n* = 5, **P < 0.01, by one-way ANOVA with Tukey correction. **(J)** The IL-6 level of HDFs implanted in different hydrogels after NIR-irradiation. *n* = 5, by one-way ANOVA with Tukey correction

Gram-positive *S. aureus* and *MRSA* and Gram-negative *E. coli* and *P. aeruginosa* were used to study the antibacterial activities of hydrogels [39]. The GDY-containing groups exhibited outstanding antibacterial properties against *S. aureus* and *E. coli*, which are the most common bacteria in diabetic wound infections. Surprisingly, the killing rate of PEGDA-GDY/G@TGFβ hydrogel against *P. aeruginosa* was 74.99 ± 6.52% and the killing rate of *MRSA* by PEGDA-GDY/G@TGFβ hydrogel is 78.51 ± 9.91%. The overall results verified that GDY-incorporated hydrogels could achieve antibacterial effects after NIR-irradiation in 30 s (Fig. 4A–E).

Inhibition zone tests were performed to further assess the antibacterial effects of the hydrogels [40]. The diameters of the inhibition zones of PEGDA toward the *S. aureus* group after NIR irradiation were much smaller than those of the other groups (Fig. 4F and Fig. S9). However, the inhibition zones for *E. coli, P. aeruginosa*, and *MRSA* did not exhibit significant differences, which might correlate with the differences in the proliferation and membrane structures of the various bacteria [41].

Moreover, effective photothermal antibacterial therapy usually requires higher temperatures (> 60 °C), which are generally higher than physiological temperatures (37 °C) and inevitably cause local thermal damage to normal tissues and cells around the wound [42]. In this research, the temperatures were controlled under 50 °C, and the antibacterial effects might be associated with potential oxidative stress responses of GDY nanoparticles after NIR-irradiation. To confirm that GDY increased the photothermal antibacterial effects, the pure temperature rise via heating was prepared, and the temperature variation was similar to that of NIR-irradiation (Fig. S10A). The controlled trial indicated that the antibacterial effects of GDY-dependent photothermal effects were significantly better than those of the pure temperature rise (Fig. S10B).

The underlying mechanism of antibacterial properties of photothermal effects at safe temperature was further surveyed. It has been reported that graphene oxide nanoparticles could enhance antibacterial efficiency *via* damage to the bacterial membrane and release of ROS; thus, we inferred that the antibacterial effects at a safe temperature of GDY-incorporated hydrogels might have similar properties [43, 44]. The amount of ROS in GDY-incorporated hydrogels was much higher than those of the other groups, and the cell membrane integrity of *S. aureus* in GDY-incorporated hydrogels decreased after NIR-irradiation, which potentially enhanced the antibacterial effects of PEGDA-GDY/G@TGFβ hydrogel (Fig. 4G and H). One reason for the difficulty in healing diabetic wounds is the over-activation of ROS [45, 46]. Therefore, we further assessed whether the amount of ROS generated by GDY nanoparticles after NIR-irradiation would cause cell damage. The generated ROS did not interfere with the cell activities of HDFs and it did not activate oxidative stress-related signaling pathways to cause negative effects (Fig. 4I and J). This offered more evidence that relevant changes in antibacterial properties did not negatively affect the biocompatibility of hydrogels.

### Cell biocompatibility and migration of PEGDA-GDY/G@TGFβ hydrogels

**Fig. 5:**
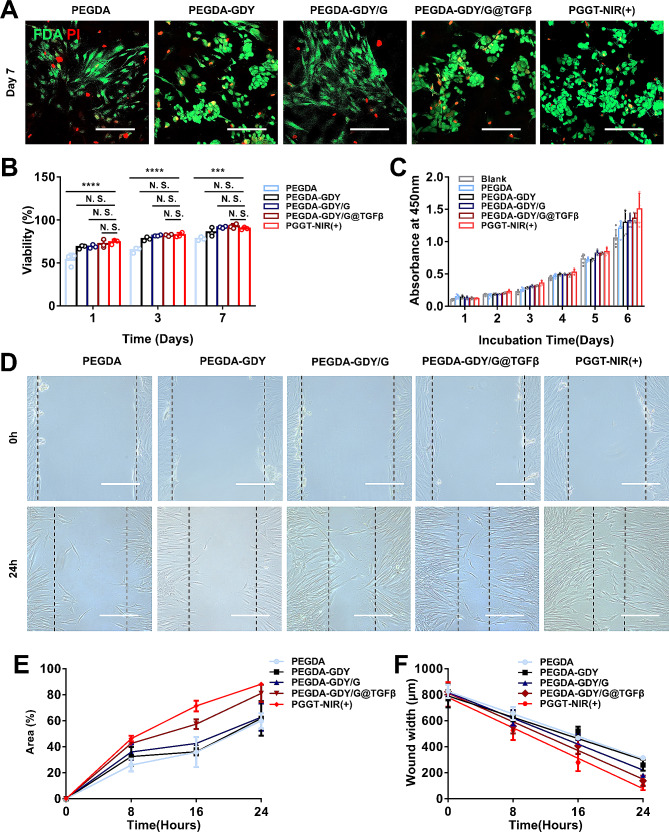
In vitro cell biocompatibility of different hydrogels. **(A)** HDFs implantation in hydrogels with FDA (green)/PI (red) staining at 7 days. Scale bar, 200 μm. **(B)** Viability of HDFs plotted by calculation of the results of biocompatibility experiments. *n* = *3*, ****P < 0.0001, ***P < *0.001*, by two-way ANOVA with Tukey correction. **(C)** The absorbance at 450 nm for CCK8 assay of different hydrogels. *n* = 5. **(D)** The images of scratch assays. Scale bar, 200 μm. Black dash line: the edge of scratch. **(E)** The percent of closure area at different time points. *n* = *3. ***(F)** The scatter plot with linear regression based on the width of the wound over time for different hydrogels. *n* = 3

The biocompatibility of hydrogels was assessed using FDA/PI staining of implanted HDFs. The cell viability in the PEGDA group after cell implantation was lower than that in the other groups, whereas the cell viability in the other groups with GDY NPs was not significantly different (Fig. 5A–B and Fig. S11). These results might be related to two reasons. On the one hand, the mixture of GDY nanoparticles was proved to improve the cell adhesion of biomaterials [22]. On the other hand, the addition of GDY could change the surface pore sizes of hydrogels and these pore sizes might be beneficial for cell infiltration and proliferation [47]. Furthermore, the addition of GDY and NIR irradiation did not significantly harm the viability of HDFs according to a cell counting kit-8 (CCK8) assay (Fig. 5C). This is an important result since the biosafety of two-dimensional nanomaterials and photothermal effects are important parameters for biomedical applications [22].

Scratch assays were performed to evaluate the effects of the hydrogels on cell migration during wound healing. The wound widths of PEGDA-GDY/G@TGFβ and PGGT-NIR (+) groups were much higher than those of the other groups after 16 and 24 h (Fig. 5D and Fig. S12). The percentage of wound closure of the PGGT (+) group (88.16 ± 1.40%) at 24 h was much higher than that of the other groups results (Fig. 5E). Width–time curves were plotted to further test the wound closure rates of the materials (Fig. 5F). Linear regression was used to calculate the rate of closure. The rate of closure of the PGGT (+) group (36.20 μm/h) was higher than that of the PEGDA-GDY/G@TGFβ group (32.25 μm/h).

### Endothelial cell adhesion and vascular formation of PEGDA-GDY/G@TGFβ hydrogels

**Fig. 6:**
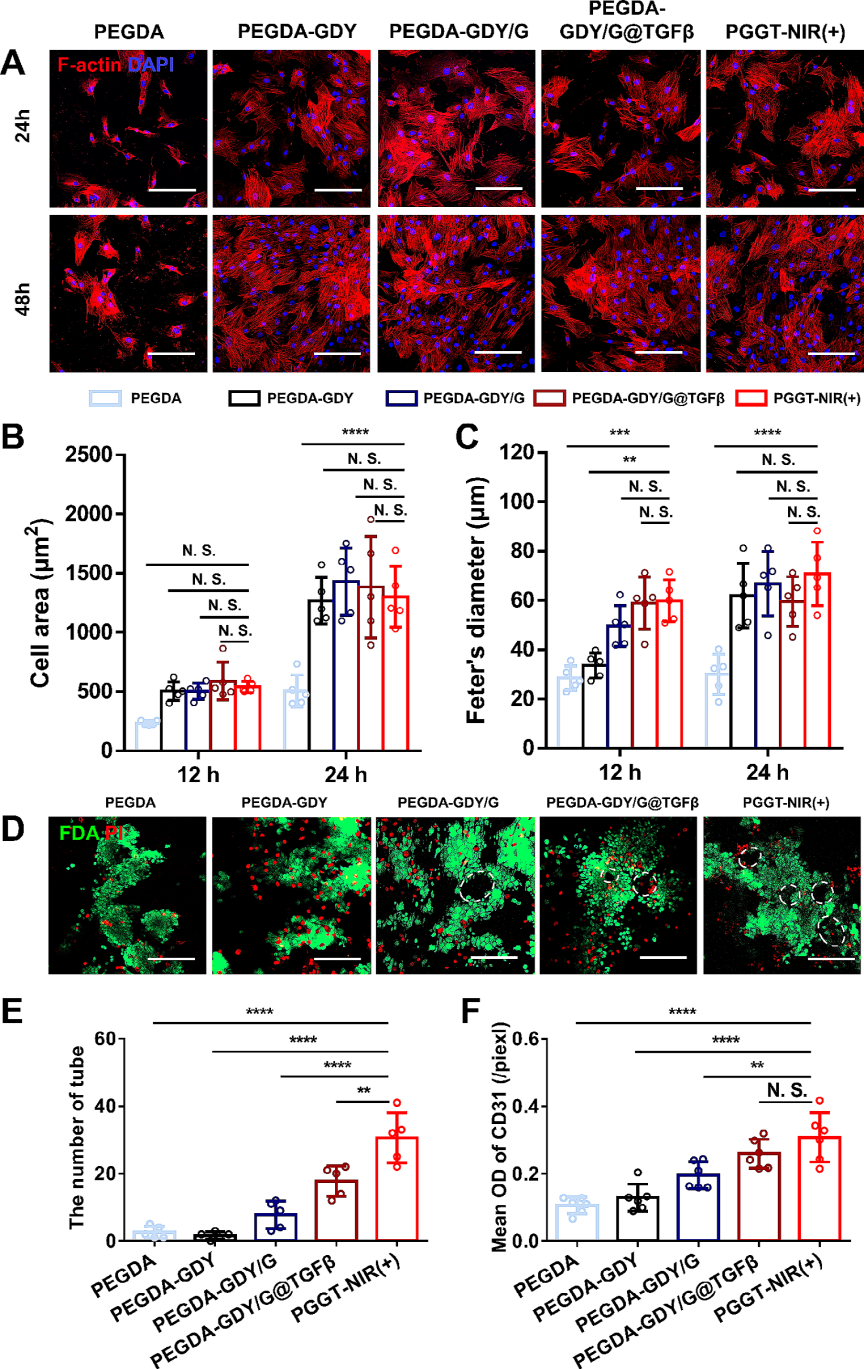
The promotion of angiogenesis properties of different hydrogels in vitro. **(A)** F-actin staining images of HUVECs implanted in different hydrogels. **(B)** Cell area and **(C)** Feter’s diameter of different hydrogels was plotted at the time points of 24 h and 48 h. *n* = *5*, ****P < *0.0001*, ***P < *0.001*, **P < *0.01*, by two-way ANOVA with Tukey correction. **(D)** FDA/PI staining of tube formation experiments at 8 h. Scale bar, 200 μm. White dash line: tubular structure. **(E)** The number of tubes in tube formation experiments was calculated. *n* = *5*, ****P < *0.0001*, **P < *0.01*, by one-way ANOVA with Tukey correction. **(F)** The mean OD values of CD31 (/piexl) were analyzed. *n* = *6*, ****P < *0.0001*, **P < *0.01*, by one-way ANOVA with Tukey correction

Blood vessel regeneration is an important factor in diabetic wound healing [48]. Therefore, angiogenic properties were tested in vitro. Human umbilical vein endothelial cells (HUVECs) were implanted into the hydrogels, and F-actin was stained to measure the adhesion of endothelial cells (ECs). The number of adherent HUVECs containing GDY NPs was greater than that in the PEGDA group (Fig. 6A). It was previously confirmed that GDY NPs enhanced cell adhesion. In addition, the HUVECs in PEGDA-GDY/G@TGFβ and PGGT-NIR (+) groups exhibited cell spreading, and actin filaments were well organized, indicating that the release of TGF-β was beneficial for EC proliferation (Fig. 6B-C).

Tube formation experiments were performed to study the promotion of angiogenesis in the hydrogels. Staining showed that a tubular structure was observed in the PEGDA-GDY/G @TGFβ and PGGT-NIR (+) groups, but not in the PEGDA and PEGDA-GDY groups (Fig. 6D), which was verified quantitatively (Fig. 6E). This indicated that the TGF-β in the hydrogels may enhance vascular regeneration. Additionally, the function of ECs was vital for the reconstruction of vessels, as reflected by CD31 staining. The optical density (OD) of the CD31 staining in the groups loaded with TGF-β was much higher than that in the other groups (Fig. 6F). The plasticity of ECs is mediated by various cytokines, including TGF-β and vessel formulation was involved in TGF-β-regulated SMAD signaling [49, 50]. Our research further verified this biological activity and its potential applications in the treatment of chronic wounds.

### Investigating PEGDA-GDY/G@TGFβ hydrogels in a diabetic wound mouse model

**Fig. 7:**
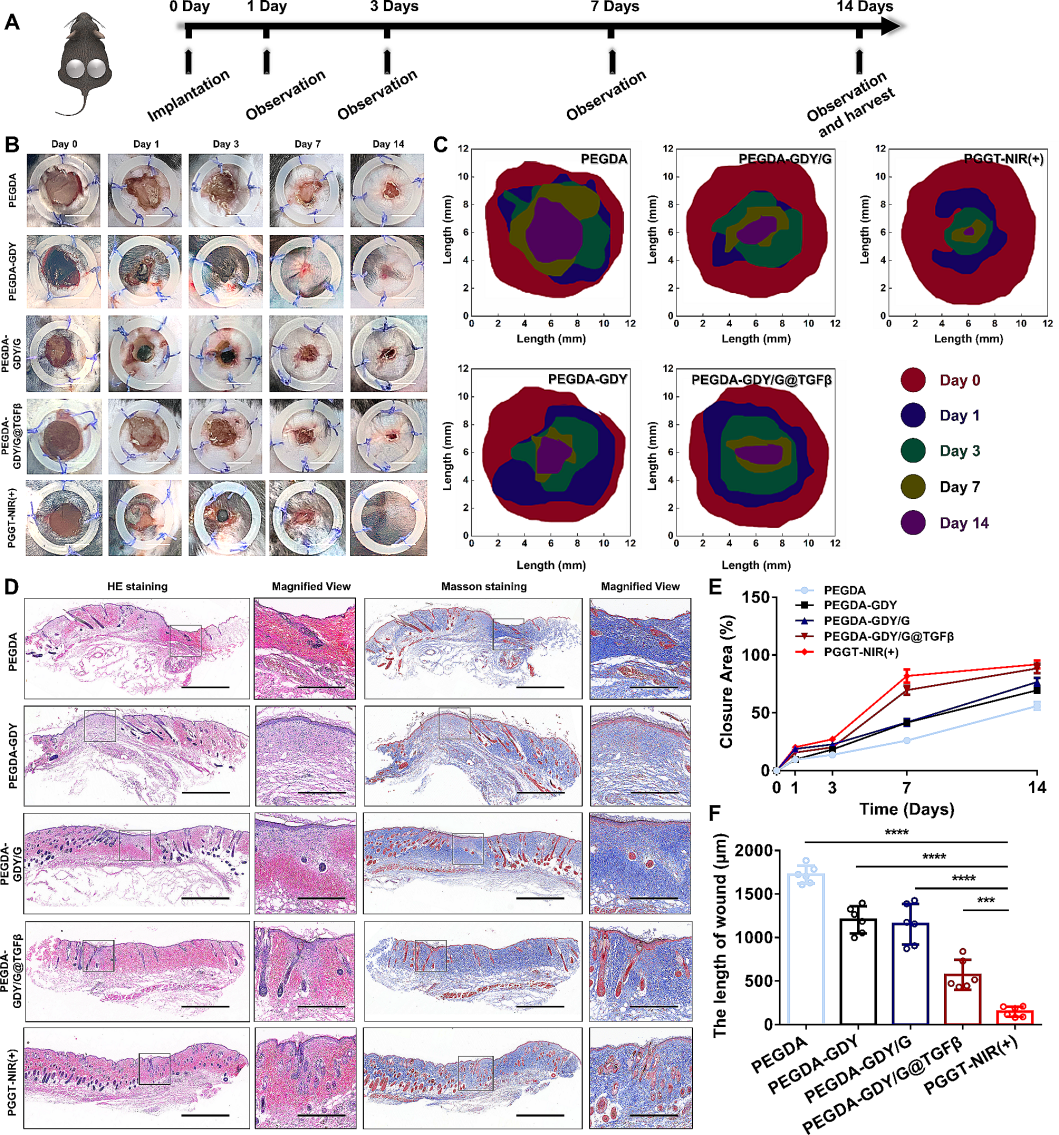
In vivo implantation of hydrogels on wounds of diabetic mice. **(A)** Schematic demonstrating the timeline of different hydrogel treatments. **(B)** Photographs of the wound after treatment with different hydrogels. Scale bar, 10 mm. **(C)** The projections of the wound at different time points after treatment with different hydrogels. **(D)** HE staining images of harvested wound tissues at 14 days. Scale bar, 500 μm, 300 μm for magnified view. **(E)** The percent of the closure area of wound after treatment with different hydrogels measured by macro views. *n* = 5. **(F)** The length of the wound after treatment with different hydrogels measured by HE staining images. *n* = *6*, ****P < *0.0001*, ***P < *0.001*, by one-way ANOVA with Tukey correction

The hydrogels were implanted into diabetic wound mouse models to study the therapeutic effects of the PEGDA-GDY/G@TGFβ hydrogels. The timeline of the wound-healing experiments is shown in Fig. 7A. Macroscopic views of the wounds in the different groups were captured on days 0, 1, 3, 7, and 14 (Fig. 7B). The wounds in the PGGT-NIR (+) group nearly disappeared, whereas defects remained in the PEGDA, PEGDA-GDY, and PEGDA-GDY/G groups. Figure 7C was generated according to the macro views for a clear visualization of the wound changes. The wound-healing patterns of PEGDA-GDY/G@ TGFβ and PGGT-NIR (+) before day 3 were like those of the other groups (Fig. 7E). In the following days, the wound-healing capacities of PEGDA-GDY/G@TGFβ and PGGT-NIR (+) were better than those of the other groups. The closure rate of the PGGT-NIR (+) group (91.96 ± 3.08%) was higher than that of the PEGDA-GDY/G@TGFβ group (88.44 ± 3.83%) on day 14. Notably, the PEGDA-GDY and PEGDA-GDY/G groups showed better wound healing than the PEGDA group, which may be related to the antibacterial effects of the GDY NPs.

For the histological analysis, the implanted hydrogels were harvested on day 14 and stained with hematoxylin and eosin (H&E), Masson’s trichrome, CD31, and immunofluorescent staining. The H&E and Masson’s trichrome staining revealed different degrees of extracellular matrix deposition (Fig. 7D). The collagen and fibrins of the unhealed wound areas of the PGGT-NIR (+) group were like those in the normal area, while those of the other groups were arranged in a disorderly fashion. Moreover, the lengths of the wounds were analyzed (Fig. 7F) and the results were consistent with those of the wound areas. The epithelia of the PEGDA-GDY/G@TGFβ (30.92 ± 6.65 μm) and PGGT-NIR (+) groups (36.77 ± 4.65 μm) were thicker than those of the other groups (Fig. S13). The wound-healing rate was the highest in groups loaded with TGF-β, consistent with previously reported findings that TGF-β could promote cell migration in vivo [51].

**Fig. 8:**
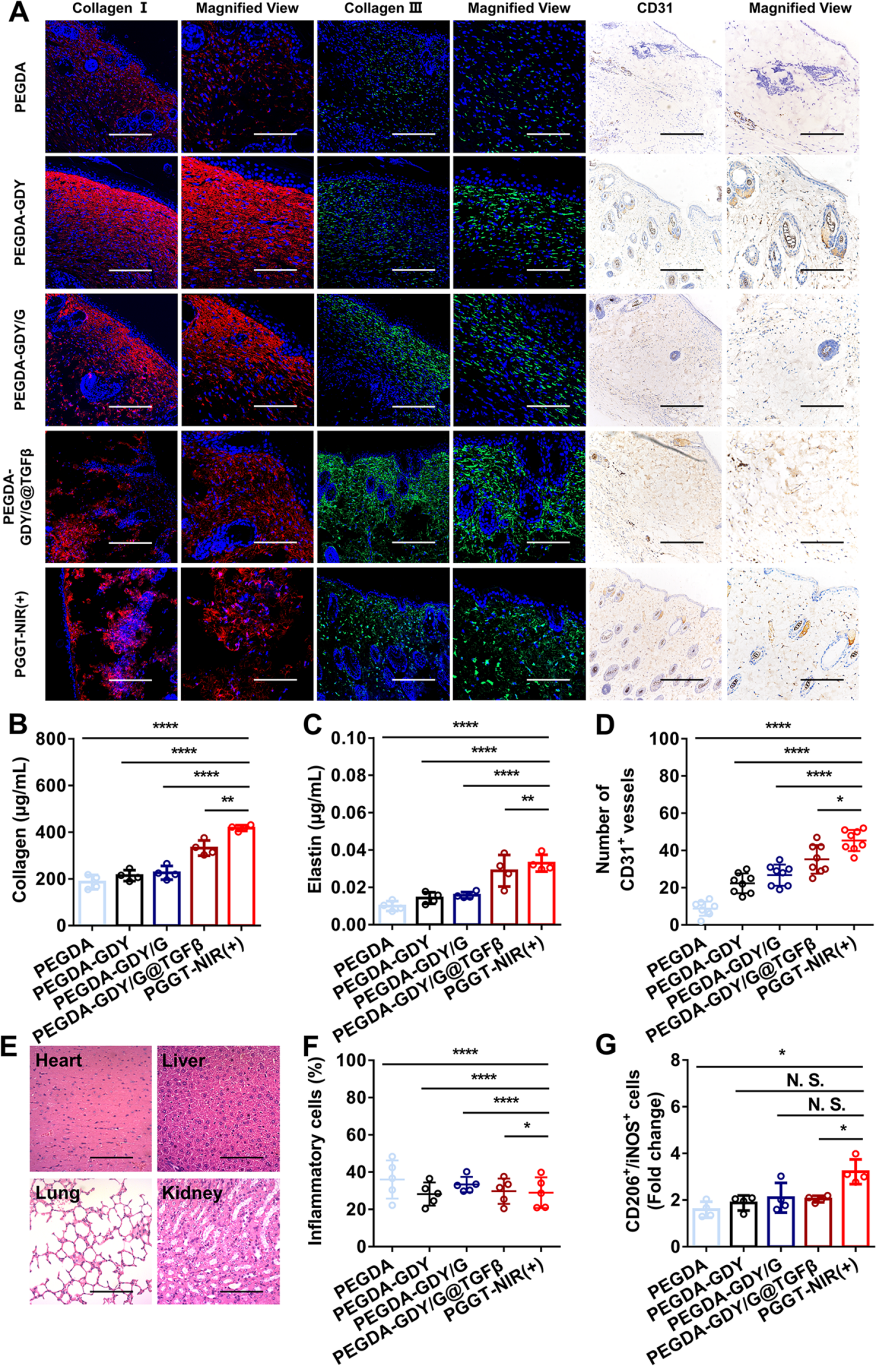
In vivo histological assessment of hydrogels on diabetic wound healing. **(A)** Immunofluorescence staining of collagen I (red), collagen III (red) and immunohistochemical staining of CD31 of wounds after treatment with different hydrogels at the time point of 14 days. **(B)** Collagen and **(C)** elastin content in harvested wounds at the time point of 14 days. *n* = 4. ****P < 0.0001, ***P < 0.001, **P < 0.01, by one-way ANOVA with Tukey correction. **(D)** The number of CD31 + vessels per 0.01 mm^2^ in the wound section. *n* = *8*, ****P < 0.0001, *P < 0.05, by one-way ANOVA with Tukey correction. **(E)** HE staining of organs after treated with PGGT-NIR (+) group after 14 days. Scale bar, 200 μm. **(F)** The ratio of CD206 + cells and iNOS + cells in wound treated with different hydrogels after 14 days. *n* = 5, by one-way ANOVA with Tukey correction. **(G)** The inflammatory cells in wounds treated with different hydrogels after 14 days. *n* = 4, *P < 0.05, by one-way ANOVA with Tukey correction

Tissue regeneration (particularly of the extracellular matrix) is important for wound healing [52]. The deposition of collagen I results in scar formation, whereas that of collagen III is beneficial for wound healing [4]. As shown in Fig. 8A, collagen I in the PEGDA-GDY/G@TGFβ and PGGT-NIR (+) groups did not show excessive proliferation, whereas the other groups (especially PEGDA-GDY) exhibited collagen I hyperplasia. Additionally, the proliferation of collagen III in the PGGT-NIR (+) group was greater than that in the other groups, and PEGDA barely deposited any collagen III. The PGGT-NIR (+) group stimulated the regeneration of the extracellular matrix (collagen and elastin) in the skin wounds and performed better than the other groups (Fig. 8B-C).

Angiogenesis is another important factor that influences diabetic wound healing [53]. CD31 immunohistochemical staining was performed to assess the ability of the hydrogels to promote vessel generation [11, 53]. The number of CD31 + vessels per 0.01 mm^2^ in the PGGT-NIR (+) group (45.375 ± 5.03) was much higher than that in the PEGDA-GDY/G@TGFβ group (35.25 ± 6.83) (Fig. 7A and D). The number of CD31 + vessels in the PEGDA-GDY/G@TGFβ group was significantly higher than that in the other groups, indicating the potential ability of TGF-β to promote revascularization.

Nanoparticle biosafety is of vital importance in the clinical applications of nanomedicine and the biosafety of the nanosystem in this study was closely monitored [54]. There was no significant NP residue or metabolites in the tissues of the heart, liver, lung and kidney (Fig. 8E). There was no significant difference in the proportion of inflammatory cells in the hydrogels of each group (Fig. 8F). This demonstrated that the GDY nanosystem in this study either did not cause substantive damage to vital organs or that the nanoparticles rarely entered the blood circulation, having only local effects in the wound. Either possibility indicated good biosafety of the hydrogel for in vivo applications. The blood routine examination and blood biochemistry test shown in Tables S1 and S2 further verified this conclusion. Moreover, the response of monocytes/macrophages to nanoparticles during tissue regeneration is important [55]. CD206 is a marker of anti-inflammatory and proregenerative macrophages and inducible nitric oxide synthase (iNOS) is a marker of proinflammatory macrophages [56]. The ratio of CD206/iNOS in the PGGT-NIR (+) group was higher than that of the PEGDA group (Fig. 8G). This indicated that the PEGDA-GDY/G@ TGFβ hydrogels provided a better immune microenvironment for wound healing.

## Discussion

In this study, we developed a PEGDA-GDY/G@TGF-β hydrogel as a wound dressing and investigated its use in diabetic wound healing. The core-shell type is commonly included in smart materials to achieve the “ON/OFF” function of the stimulus-response [57]. In our study, the smart response was dependent on gelatin that turned from a gel state to a fluid state at the temperature above 30 °C. This prominent feature has been widely used in sacrificial materials and physical and chemical modifications [58]. On one hand, we can use the charge modifications to shift the charge on GDY NPs from negative to positive to load TGF-β as a drug. On the other hand, heat-induced melting of the gelatin coating during NIR irradiation makes optically controlled drug release possible. Besides, photo-sensitiveness properties of PEGDA contained in hydrogel also indicated the potential for 3D printing fabrication in further research, which will be a fabrication method better than the molding method utilized in this research [59, 60].

Graphdiyne has been explored for potential biomedical applications owing to its excellent properties and stable structure. A mixture of GDY in biomedical scaffolds was investigated to impart the free-radical scavenging ability, strengthen mechanical properties, and enhance cell adhesion [61, 62]. The surface modification of GDY nanoparticles that might change the pore sizes of scaffolds also have been found in previous and this study [22]. However, the potential of GDY NPs as drug carriers and smart materials in nanomedicine was not investigated. In this study, we developed a drug-loaded nanosystem based on GDY NPs. The hydrogels with GDY showed good biocompatibility in vitro and in vivo and were sensitive to photothermal reactions. Moreover, the rate of change of temperature of the GDY NPs was much quicker than that of graphene oxide NPs and the targeted temperature (approximately 50 °C) could be reached in seconds [63]. This property was beneficial for the stability of protein drugs, such as cell factors [64]. Most importantly, graphdiyne-incorporated hydrogels exhibited good antibacterial properties for drug-resistance bacteria such as *P. aeruginosa* and *MRSA* (killing rate > 75%). Graphdiyne NPs achieved a high rate of antibacterial effects under 49.5 °C depending on the damage to the membrane of bacteria and potential oxidative stress response and these might strengthen photothermal bactericidal effects. Consequently, the different killing effects on various bacteria correlated with the proliferation model and structure of the bacterial membranes. However, the inhibitory zone experiments cannot prove the diffusion effects of the PEGDA-GDY/G@TGFβ hydrogels on bacteria [39]. In fact, to achieve the high efficiency of sterilization at low temperatures, many efforts have been spared. Zhao et al. reported a versatile hydrogel dressing consisting of phenylboronic acid-functionalized graphene and oxadiazole-decorated quaternary carboxymethyl chitosan (QCS). The membrane permeability of QCS enhanced bacterial vulnerability, which is beneficial to photothermal therapy against *MRSA in vitro* and in vivo at temperatures of < 49.6 °C [65]. Finally, the influence of GDY NPs on ROS is worthwhile discussing. In this study, GDY nanoparticles did not possess the ability to capture ROS. Xie et al. reported a nanoradioprotector based on bovine serum albumin-modified GDY nanoparticles, which could effectively inhibit ROS-induced apoptosis signal pathway, and thus reduce gastrointestinal cell apoptosis [66]. We would like to incorporate this sort of modification into our further research plan to construct antioxidant GDY nanosystems.

TGF-β is an important cytokine in the induction of endothelial-mesenchymal transition and plays a key role in wound healing [67]. For example, TGF-β acts as a chemotactic protein of fibroblasts [13]. The expression of TGF-β receptors by fibroblasts involved in wound healing has been examined in normal and healed skin, particularly in healing scars [10, 68]. In this study, TGF-β was innovatively loaded into GDY/G NPs to be delivered in vivo and in vitro. The groups loaded with TGF-β promoted cell migration in vitro and the wound healing rate of PGGT-NIR (+) was the highest, consistent with previously reported findings [69]. Interestingly, we found that TGF-β at low concentrations had no effects on antibacterial activity and this cannot be denied that antibacterial effects of TGF-β might occur with increasing concentration. ROS production (induced by a high-glucose environment) modulates TGF-β signaling through different pathways, including the SMAD pathway [70]. Activated TGF-β may increase ROS production and suppress antioxidant enzymes, forming a vicious cycle in many fibrotic diseases [67, 71]. However, this cycle may be beneficial for the fibrosis of diabetic wounds in the early stages of the disease and angiogenesis in the later stages, which was confirmed in the cell migration tests and tube formation tests. In addition, it has been reported that platelets exosome product enriched with TGF-β could significantly promote full-thickness healing with the re-acquisition of hair follicles and sebaceous glands, which might be related to TGF-β/bone morphogenetic protein signaling pathways [11, 72]. In our study, more hair follicles are regenerated in PEGDA-GDY/G@TGFβ group than in other groups. Nevertheless, the properties and mechanics of TGF-β influencing hair follicles in diabetic wounds still demand further research. Finally, the release of TGF-β in vivo was worth measuring to clarify the sustained and controlled release on diabetic wounds, which will also be included in our future research.

## Conclusion

We designed and fabricated PEGDA-GDY/G@TGFβ hydrogels with NIR-controlled drug release and antibacterial effects. Moreover, PEGDA-GDY/G@TGFβ hydrogel exhibited the ability to promote cell migration and angiogenesis when applied as a wound dressing for diabetic wounds. This study also suggested the possibility of combining GDY and gelatin as a promising carrier for cell factors and confirmed the sensitivity of GDY NPs in photothermal effects. Most importantly, the excellent properties of PEGDA-GDY/G@TGFβ hydrogels made them suitable for further application in the treatment of wounds in clinics.

### Electronic supplementary material

Below is the link to the electronic supplementary material.

Supplementary Material 1

## Data Availability

Data is provided within the manuscript or supplementary information files.

## Abbreviations

GDY: Graphdiyne
iNOS: Inducible nitric oxide synthase
NIR: Near-infrared
NP: Nanoparticle
PDI: Polydispersity index
PEGDA: Polyethylene glycol diacrylate
ROS: Reactive oxygen species
TGF-β: Transforming growth factorβ
