# Supplementary material for: A core-shell-type nanosystem promotes diabetic wound healing through Photothermal-responsive release of transforming growth factor β
Source: J Nanobiotechnology. 2024 Jul 30;22:449. doi: 10.1186/s12951-024-02675-2 (PMC11287882; doi:10.1186/s12951-024-02675-2)
Supplement: Supplementary file 1 — Supplementary Material 1 [file 12951_2024_2675_MOESM1_ESM.pdf]

Supplementary Materials for

A Core-shell Type Nanosystem Promotes Diabetic Wound Healing Through Photothermal-Responsive Release of Transforming Growth Factor β

Jinfei Hou[1†], Junjin Jie[2,3†], Xinwei Wei[4†], Xiangqian Shen[1], Qingfang Zhao[1], Xupeng Chai[5], Hao Pang[1], Zeren Shen[1], Jinqiang Wang[4*], Linping Wu[2,6*], Jinghong Xu[1*]*

[1]Department of Plastic Surgery, the First Affiliated Hospital, Zhejiang University School of Medicine, Hangzhou, 310003, Zhejiang, China

[2]Center for Chemical Biology and Drug Discovery, Guangzhou Institute of Biomedicine and Health, Chinese Academy of Sciences, Guangzhou, 510530, China

[3]Department of Plastic Surgery, Union Hospital, Tongji Medical College, Huazhong University of Science and Technology, Wuhan, 430022, China

[4]Key Laboratory of Advanced Drug Delivery Systems of Zhejiang Province, College of Pharmaceutical Sciences, Zhejiang University, Hangzhou, 310058, China

[5]Department of Orthopedic Surgery, the Second Affiliated Hospital, Zhejiang University School of Medicine, Hangzhou, 310009, China

[6]Key Laboratory of Immune Response and Immunotherapy, Guangzhou Institutes of Biomedicine and Health, Chinese Academy of Sciences

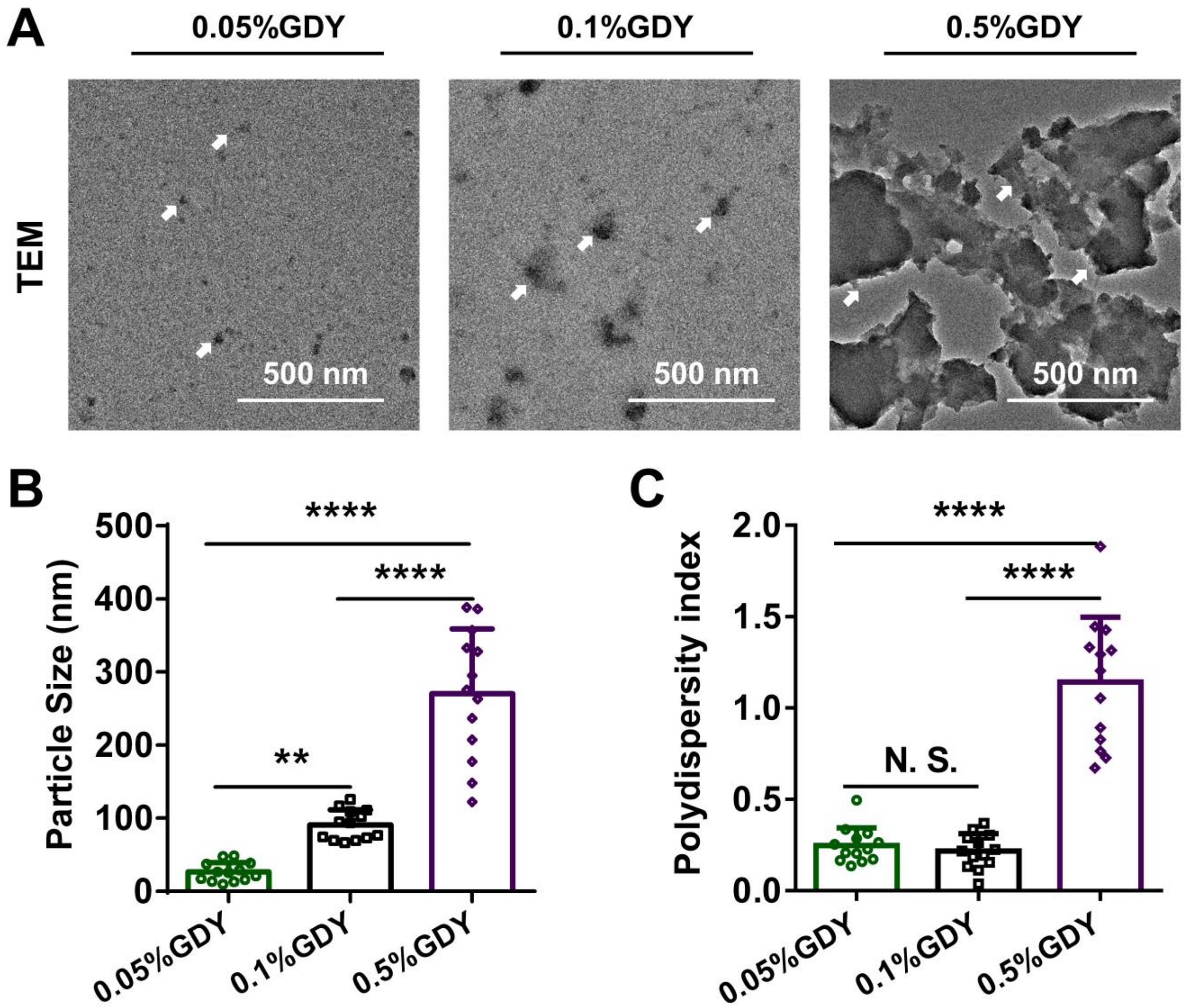

Fig. S1. Nanoparticles characterization of GDY NPs with different concentration. **(A)** TEM images, **(B)** particles size and **(C)** polydispersity index of GDY NPs with different concentration. White arrow: GDY particles. *n = 13, ****P< 0.0001, **P < 0.01,* by one-way ANOVA with Tukey correction.

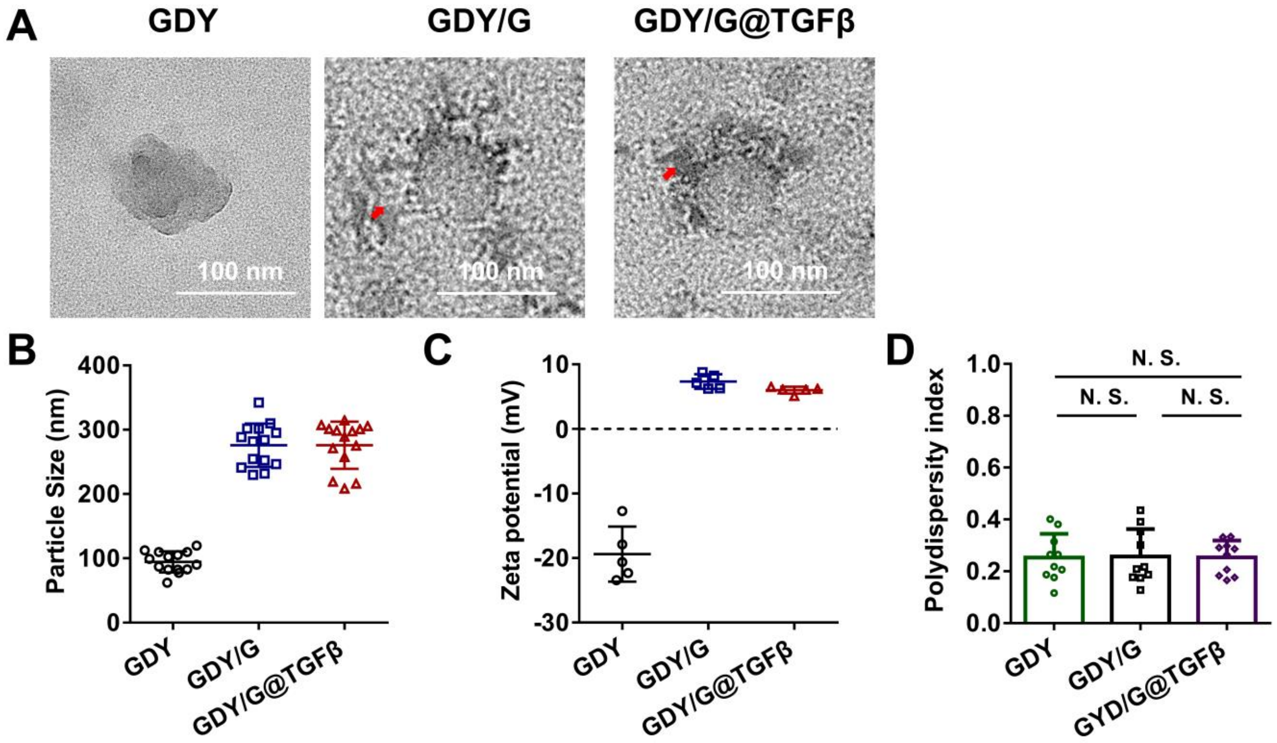

Fig. S2. (A) TEM images, **(B)** particle size and **(C)** zeta potential for different groups. Scale bar, 100 nm. Red arrow: gelatin coating. *(n = 14 for B and n = 5 for C)*. **(D)** Polydispersity index of different groups. *n = 10,* by one-way ANOVA with Tukey correction.

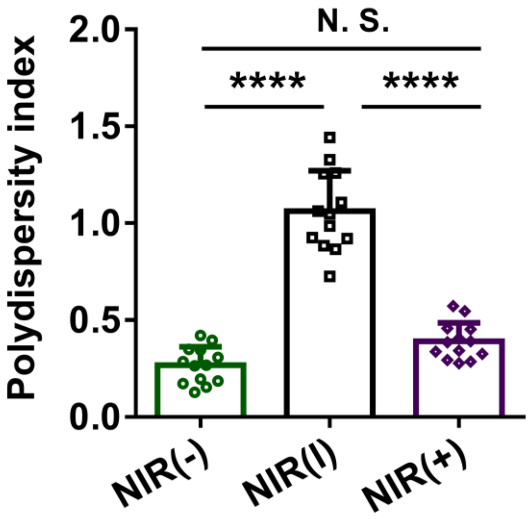

Fig. S3. Polydispersity index of PEGDA-GDY/G@TGFβ before and after NIR irradiation. NIR (-): without NIR-irradiation. NIR (|): with NIR-irradiation for 30 s. NIR (+): with NIR-irradiation for 120 s. *n = 13, ****P < 0.0001,* by one-way ANOVA with Tukey correction.

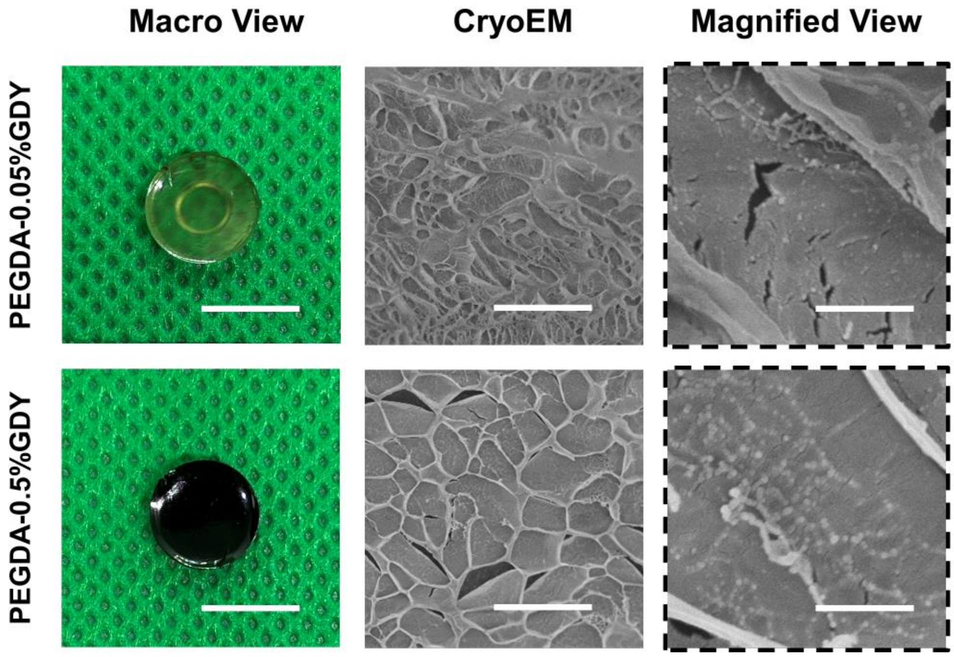

Fig. S4. Macro view and cryoEM view of different hydrogels. Scale bar: 1 μm for macro view. 50 μm for CryoEM view. 5 μm for magnified view.

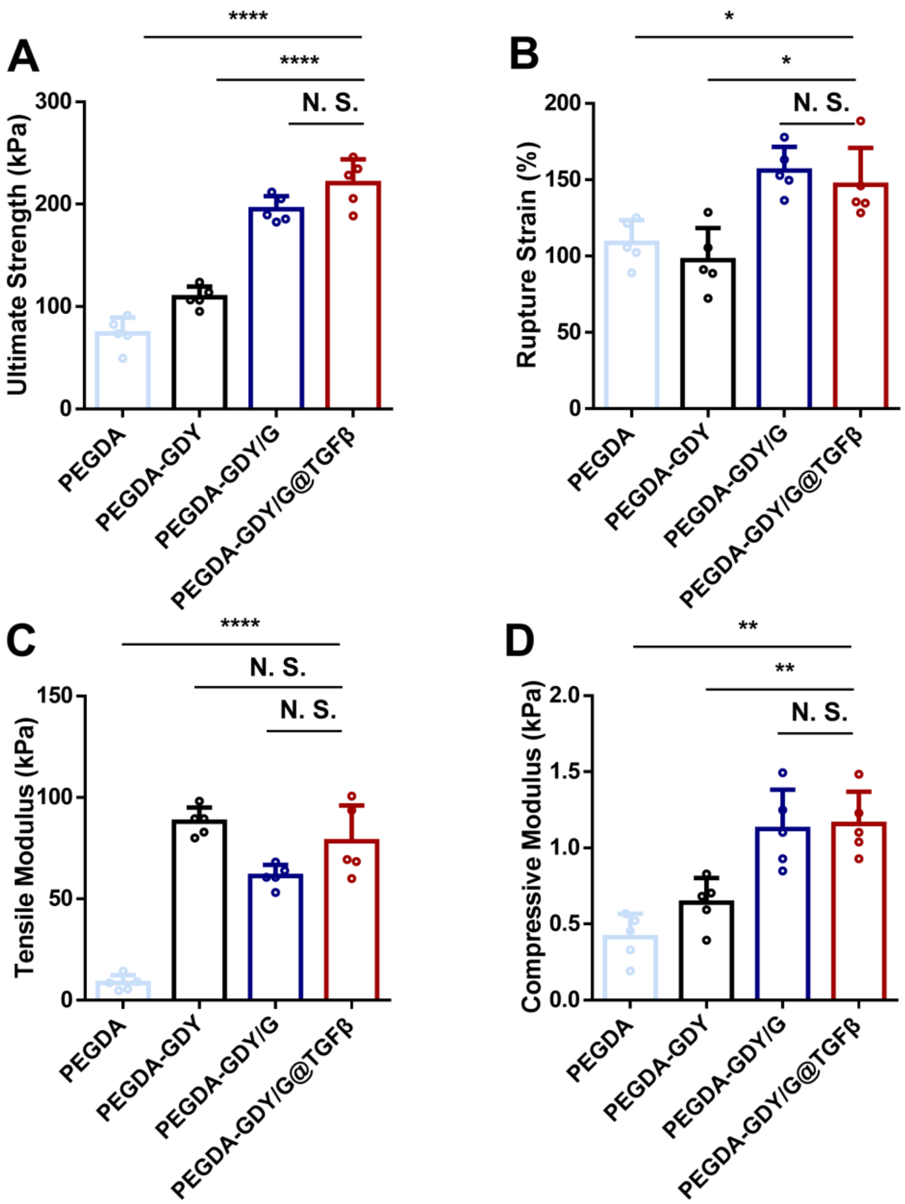

Fig. S5. Mechanical properties of different hydrogels. **(A)** The ultimate strength, **(B)** rupture strain, **(C)** tensile modulus, and **(D)** compressive modulus of different gels. *n = 5, ****P < 0.000, **P < 0.01 and **P < 0.05,* by one-way ANOVA with Tukey correction.

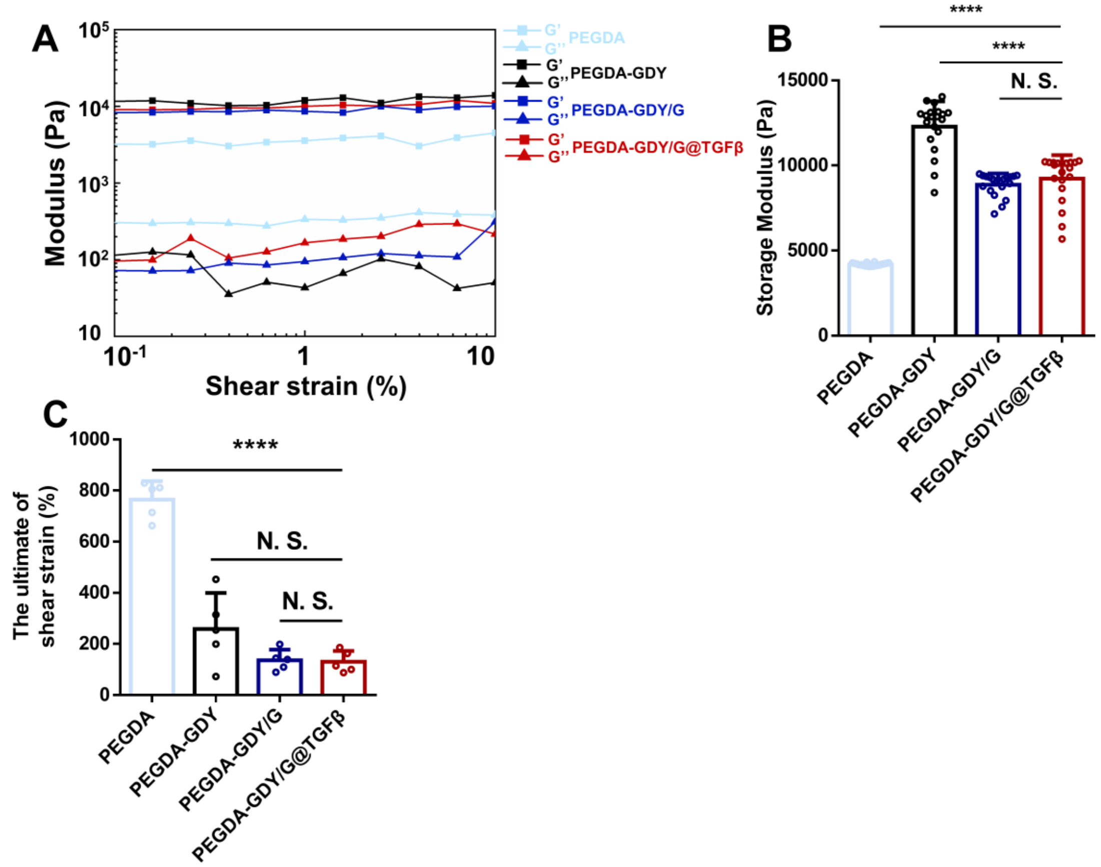

Fig. S6. Hydromechanical properties of the gels. **(A)** Frequency sweep and **(B)** storage modulus of different gels. $n = 20$, $****P < 0.0001$, by one-way ANOVA with Tukey correction. **(C)** Ultimate shear strain of different gels. $n = 5$, $****P < 0.0001$, by one-way ANOVA with Tukey correction.

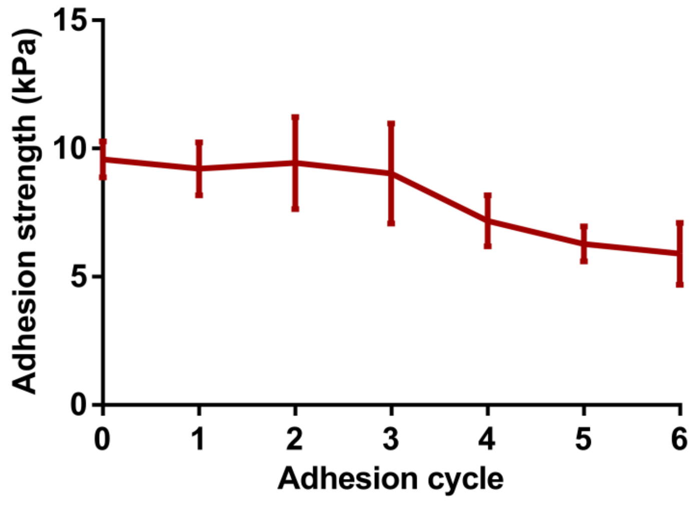

Fig. S7. Adhesion strength of PEGDA-GDY/G@TGFβ after adhesion cycles.

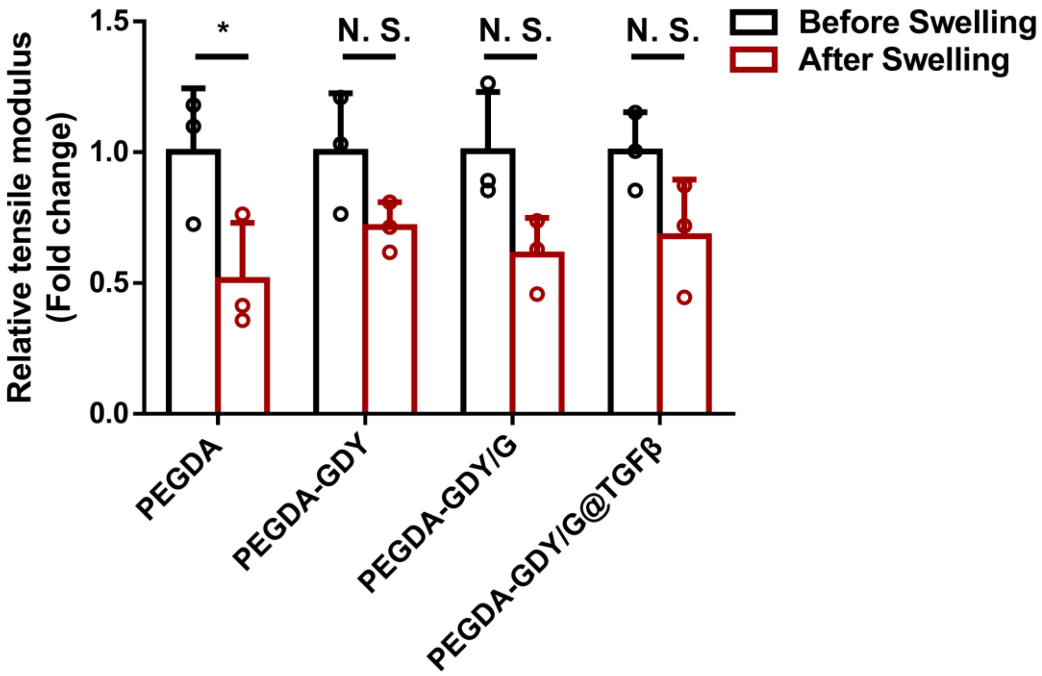

Fig. S8. Relative tensile modulus of different hydrogels before and after swelling. $n = 3$, $*P < 0.05$, by two-way ANOVA with Sidak correction.

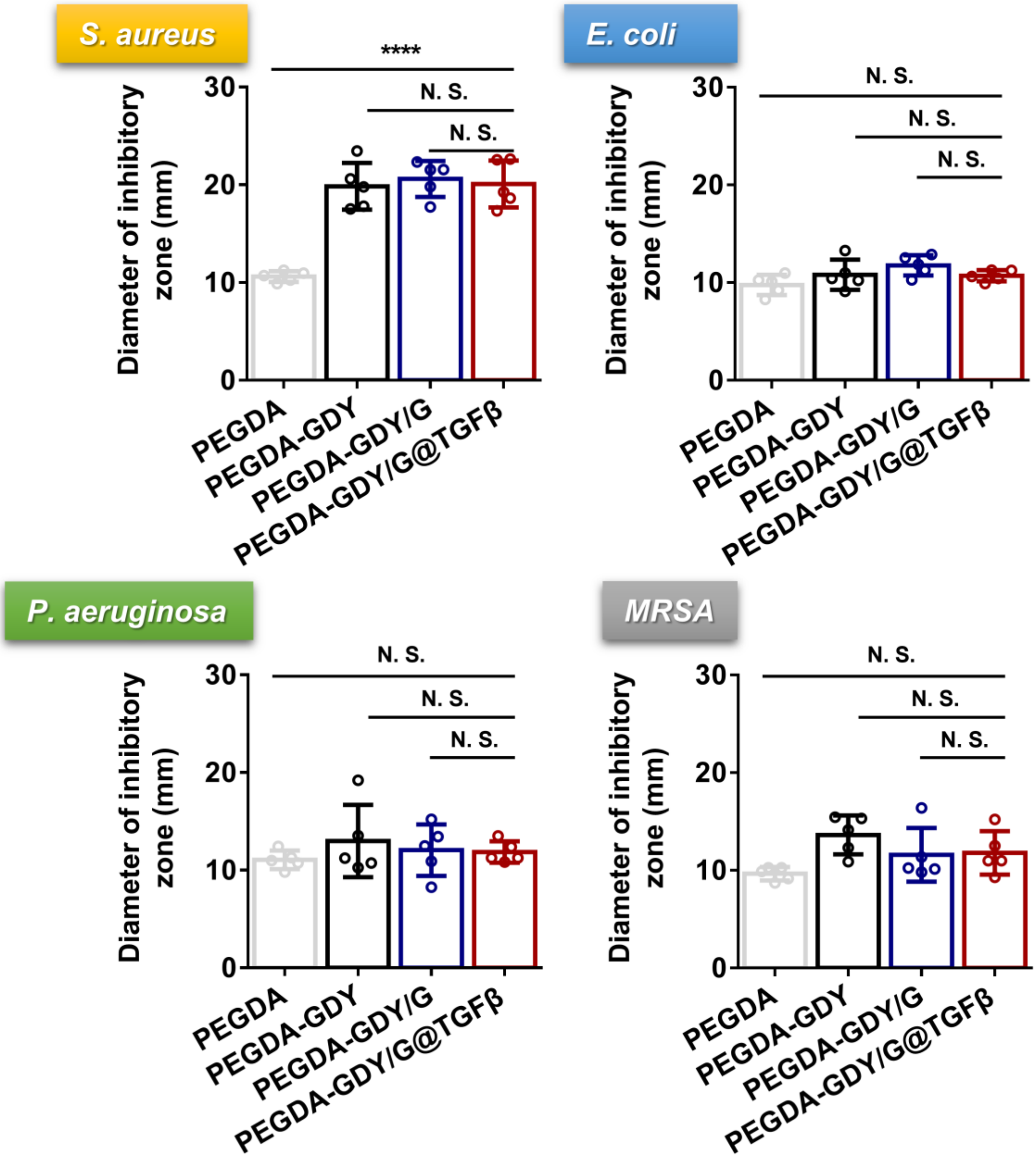

Fig. S9. The analysis of diameter of inhibitory zone for different bacteria in different hydrogels after NIR-irradiation. $n = 5$, $****P < 0.0001$, by one-way ANOVA with Sidak correction.

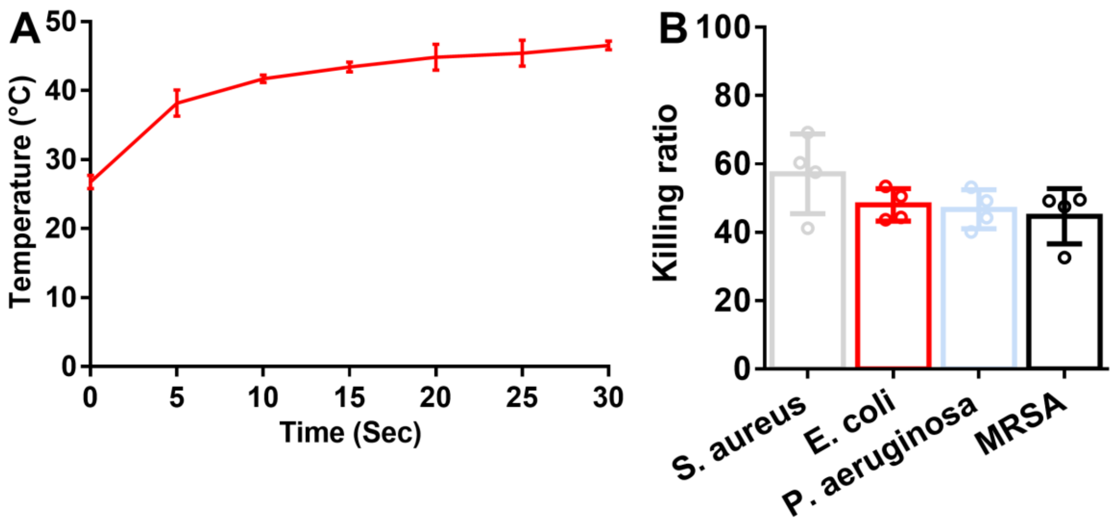

Fig. S10. (A) The time-temperature curve of heating. (B) The antibacterial effects to different bacteria. *n = 4*.

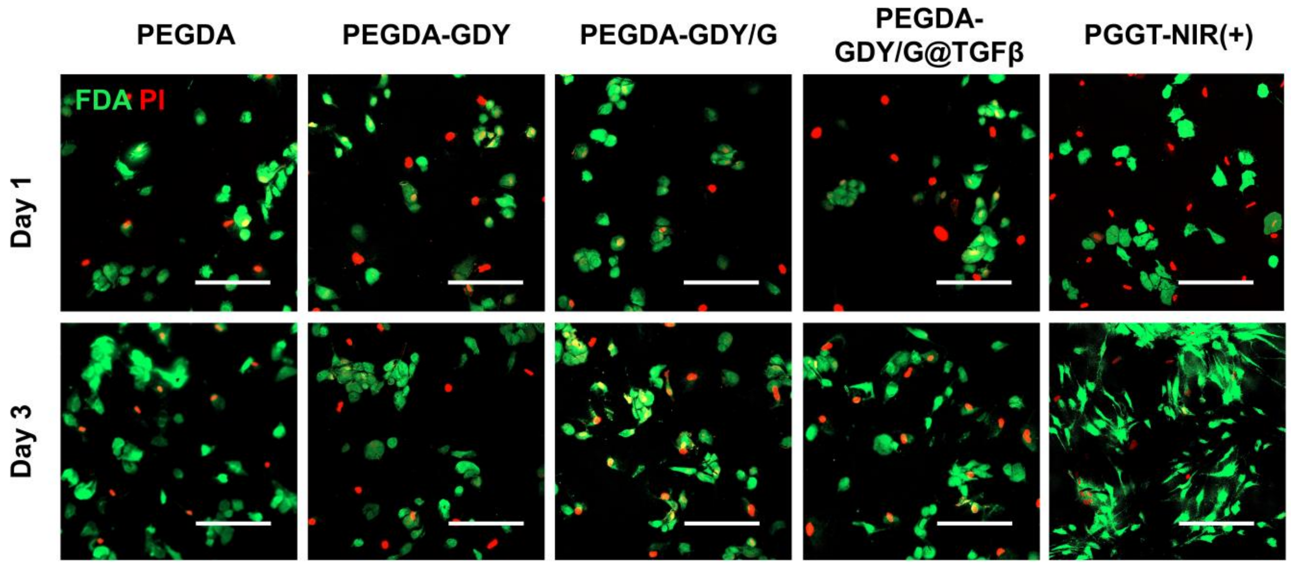

Fig. S11. FDA/PI staining of HDFs after implantation in hydrogels. Scale bar, 200 μm.

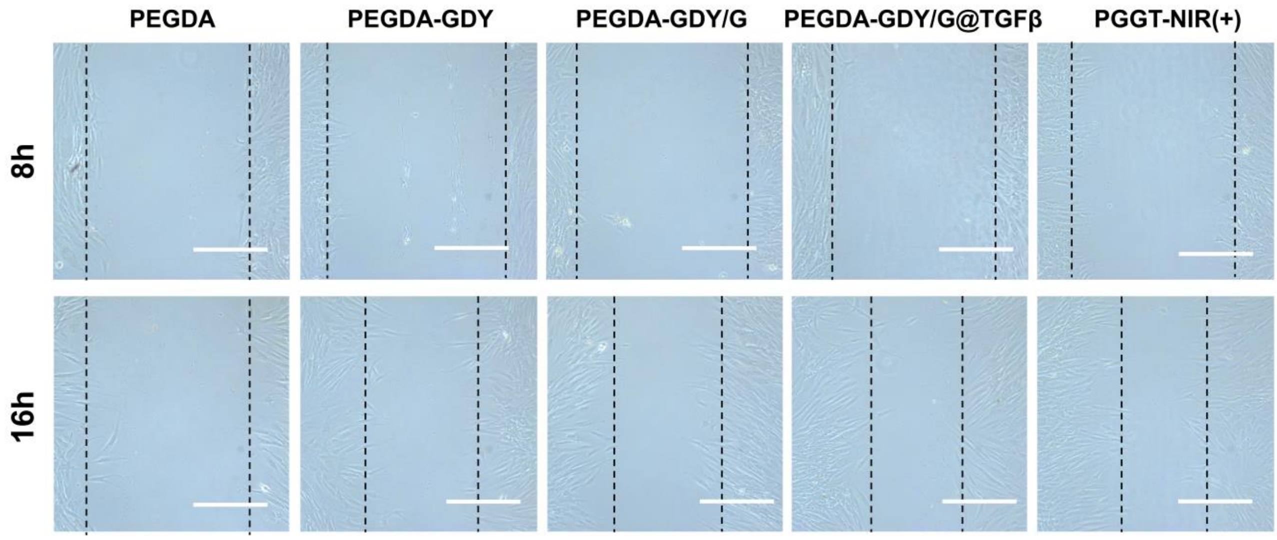

Fig. S12. The images of scratch assays. Scale bar, 200 μm. Black dash line: the edge of scratch.

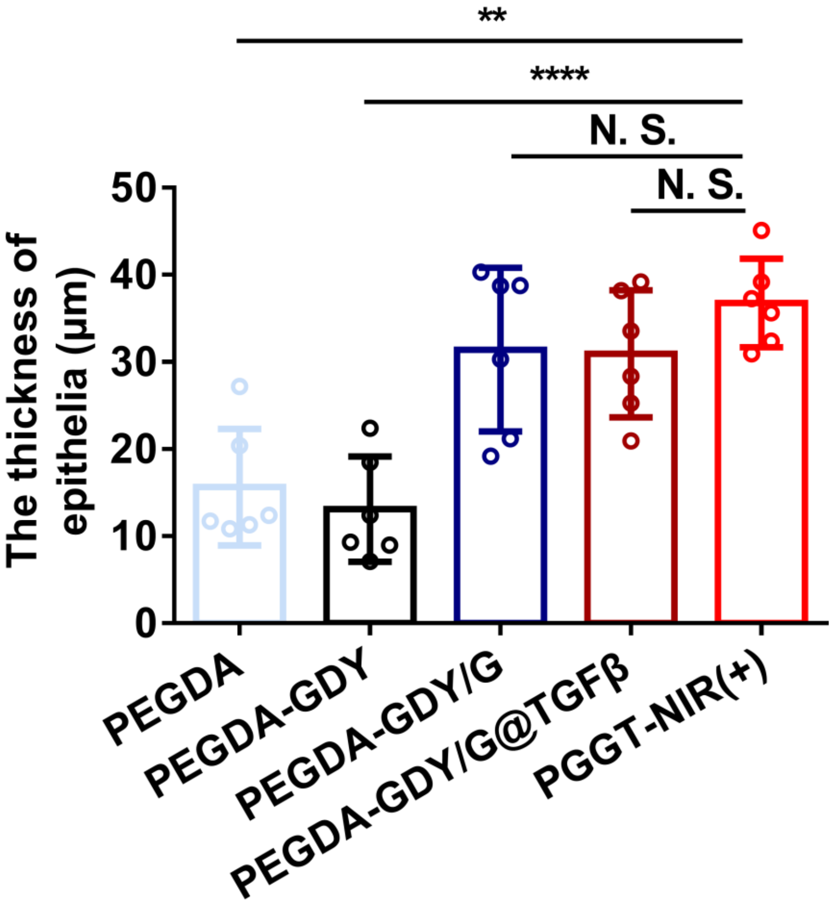

Fig. S13. The thickness of epithelia of wound in different groups. *n = 6, ****P < 0.0001, **P < 0.01,* by one-way ANOVA with Tukey correction.

Table S1. Blood routine examinations of mice post implantation *in vivo*

Liquid	WBC,10^9/L	Lymphocyte,10^9/L	Monocyte,10^9/L	Neutrophil, 10^9/L	RBC,10^9/L
Sham	21.6±6.2	5.2±3.6	1.5±0.2	10.2±1.4	9.2±0.5
PPGT	22.5±3.4	5.7±6.2	2.2±0.4	11.5±0.2	10.1±0.8

Table S2. Biochemical results of mice post implantation *in vivo*

	Blood urea nitrogen, mg/dL	Creatinine, mg/dL	Aspartate aminotransferase, U/L	Alanine aminotransferase, U/L
Sham	21.6±6.2	5.2±3.6	1.5±0.2	10.2±1.4
PPGT	22.5±3.4	5.7±6.2	2.2±0.4	11.5±0.2

Table S3 Antibodies used in immunohistofluorescence.

Primary	Catalog	Isotype	Company	Dilution	Secondary	Catalog	Company	Dilution
Collagen I	GB11022-3	Rabbit	Servicebio	1:500	Goat polyclonal to Rabbit Cy3	GB21303	Servicebio	1:300
CD206	GB13438	Rabbit	Servicebio	1:500				
iNOS	GB11119	Rabbit	Servicebio	1:500				
CD31	GB11063-2	Rabbit	Servicebio	1:1000				
Collagen Ш	GB13023-3	Rabbit	Servicebio	1:200	Goat polyclonal to Rabbit AF@488	ab150081	Abcam	1:200
